# Effect of Two or Six Doses 800 mg of Albendazole Every Two Months on *Loa loa* Microfilaraemia: A Double Blind, Randomized, Placebo-Controlled Trial

**DOI:** 10.1371/journal.pntd.0004492

**Published:** 2016-03-11

**Authors:** Joseph Kamgno, Patrick Nguipdop-Djomo, Raceline Gounoue, Mathurin Téjiokem, Annette C. Kuesel

**Affiliations:** 1 Centre for Research on Filariasis and other Tropical Diseases (CRFilMT), Yaoundé, Cameroon; 2 Faculty of Medicine and Biomedical Sciences, University of Yaoundé 1, Yaoundé, Cameroon; 3 London School of Hygiene & Tropical Medicine, London, United Kingdom; 4 Parasitology and Ecology Laboratory, Department of Animal Biology and Physiology, Faculty of Science, University of Yaoundé 1, Yaoundé, Cameroon; 5 Centre Pasteur du Cameroun, Yaounde Cameroon; 6 UNICEF/UNDP/World Bank/WHO Special Programme on Research and Training in Tropical Diseases (TDR), WHO, Geneva, Switzerland; Centers for Disease Control and Prevention, UNITED STATES

## Abstract

**Background:**

Loiasis is a parasitic infection endemic in the African rain forest caused by the filarial nematode *Loa loa*. Loiasis can be co-endemic with onchocerciasis and/or lymphatic filariasis. Ivermectin, the drug used in the control of these diseases, can induce serious adverse reactions in patients with high *L loa* microfilaraemia (LLM). A drug is needed which can lower LLM below the level that represents a risk so that ivermectin mass treatment to support onchocerciasis and lymphatic filariasis elimination can be implemented safely.

**Methodology:**

Sixty men and women from a loiasis endemic area in Cameroon were randomized after stratification by screening LLM (≤30000, 30001–50000, >50000) to three treatment arms: two doses albendazole followed by 4 doses matching placebo (n = 20), six doses albendazole (n = 20) albendazole or 6 doses matching placebo (n = 20) administered every two months. LLM was measured before each treatment and 14, 18, 21 and 24 months after the first treatment. Monitoring for adverse events occurred three and seven days as well as 2 months after each treatment.

**Principal Findings:**

None of the adverse events recorded were considered treatment related. The percentages of participants with ≥ 50% decrease in LLM from pre-treatment for ≥ 4 months were 53%, 17% and 11% in the 6-dose, 2-dose and placebo treatment arms, respectively. The difference between the 6-dose and the placebo arm was significant (p = 0.01). The percentages of participants with LLM < 8100 mf/ml for ≥4 months were 21%, 11% and 0% in the 6-dose, 2-dose and placebo treatment arms, respectively.

**Conclusions/ Significance:**

The 6-dose regimen reduced LLM significantly, but the reduction was insufficient to eliminate the risk of severe and/or serious adverse reactions during ivermectin mass drug administration in loiasis co-endemic areas.

## Author Summary

Loiasis is a big obstacle for the elimination of onchocerciasis and lymphatic filariasis in Central Africa in areas where loiasis is endemic. In these areas, some subjects who are heavily infected (microfilaraemia > 30 000 microfilariae/ml blood) can develop severe and serious adverse reactions to ivermectin. In rare cases, these have been fatal. A way of preventing these reactions could be to administer a treatment that decreases the microfilareamia in all subjects below the risk level before mass treatment with ivermectin. Building on results of previous studies, this randomised placebo-controlled trial evaluated the efficacy and safety of two or six doses of albendazole administered every two months on the microfilaraemia of *Loa loa*. Six doses led to a decrease in microfilaraemia by at least 50% for at least four months in 53% of participants. However, it did not reduce the microfilaraemia below the risk level in all participants. Therefore, this regimen has not sufficient efficacy to prevent severe adverse reactions to ivermectin.

## Introduction

Loiasis is a parasitic infection endemic in the African equatorial rain forest areas, caused by the filarial nematode *Loa loa*. It is estimated that at least 14.4 million people live in loiasis endemic areas [1]. Clinical manifestations include chronic intense itching and transient localized edema [2]. The chronic eosinophilia observed in infected individuals has been associated with endomyocardial fibrosis and related heart failure [3, 4]. Spontaneous encephalitis has been described in some heavily infected patients [5]. As for other neglected tropical diseases, the limited geographic distribution of loiasis and the fact that it mainly affects poor rural populations have limited research on this disease [6].

There is currently no safe and effective treatment. Diethylcarbamazine is effective against the larvae and adult *Loa loa* [7], but can cause serious adverse reactions (SAR, for definition see Table 1 [8]), such as meningoencephalitis, which can be fatal [9, 10]. The rapid and massive effect of ivermectin on *Loa loa* blood-dwelling microfilariae can also lead to severe adverse drug reactions (ADRs, Table 1 [8]), including SARs such as a cerebral malaria-like encephalopathy which requires hospitalization, can lead to coma and is often fatal [11, 12]. The mechanisms of these adverse reactions are not well understood. Three mechanisms have been postulated, including (1) the obstruction of the cerebral microcirculation in consequence of massive amounts of paralyzed or dead microfilariae, (2) the penetration of live microfilariae into the brain tissue following treatment, and (3) the inflammatory processes in the brain resulting from massive release of antigen from dead microfilariae [13].

**Table 1:** Adverse event terminology and definitions.

Term (Abbreviation)	Definition [8]
Adverse event (AE)	Any untoward medical occurrence in a patient or clinical investigation subject administered a pharmaceutical product and which does not necessarily have to have a causal relationship with this treatment.
Adverse drug reaction (ADR)	For marketed medicinal products: A response to a drug which is noxious and unintended and which occurs at doses normally used in man for prophylaxis, diagnosis, or therapy of disease or for modification of physiological function.
	For new medicinal products or new uses of approved products: all noxious and unintended responses to a medicinal product related to any dose should be considered adverse drug reactions.
Serious Adverse Event (SAE), Serious Adverse Reaction (SAR)	A serious adverse event (experience) or reaction is any untoward medical occurrence that at any dose:
	- results in death,
	- is life-threatening,
	*NOTE*: The term "life-threatening" in the definition of "serious" refers to an event in which the patient was at risk of death at the time of the event; it does not refer to an event which hypothetically might have caused death if it were more severe.
	- requires inpatient hospitalisation or prolongation of existing hospitalisation,
	- results in persistent or significant disability/incapacity, or
	- is a congenital anomaly/birth defect.
	Important medical events that may not be immediately life-threatening or result in death or hospitalisation but may jeopardise the patient or may require intervention to prevent one of the other outcomes listed in the definition above should also usually be considered serious.
	Note: The terms 'serious' and 'severe' are not synonymous. The term "severe" is often used to describe the intensity (severity) of a specific event (as in mild, moderate, or severe myocardial infarction); the event itself, however, may be of relatively minor medical significance (such as severe headache). This is not the same as "serious," which is based on patient/event outcome or action criteria usually associated with events that pose a threat to a patient's life or functioning. Seriousness (not severity) serves as a guide for defining regulatory reporting obligations.

The current evidence suggests that the risk of ADRs post-ivermectin is positively correlated with *Loa loa* microfilaraemia (LLM). The relatively low number of participants with known pre-treatment LLM and adverse event data available from prospective studies [14, 15] together with the likely underreporting of SARs and non-serious ADRs and the lack of pre-treatment LLM values from mass treatment with ivermectin [16], do not permit a definition of the minimum pre- treatment LLM that puts subjects at risk for development of severe ADRs and/or SARs. The study by Gardon and colleagues showed that a pre-treatment LLM ≥8100mf/ml is associated with significantly increased risk of 'marked reactions' (defined by the investigators as reactions accompanied by functional impairment requiring assistance for several days) as well as 'serious reactions' (SR, including (a) non-neurological reactions associated with functional impairment which required at least a week of full-time assistance to resume normal activities and (b) reactions with objective neurological signs with hospital admission). It was estimated that participants with pre-treatment LLM ≥50000 mf/ml were 1000 times more likely to develop SR and that those with LLM >30000 were more than 200 times more likely to develop SR than non-infected participants [15].

Onchocerciasis and lymphatic filariasis (LF) are two of the 17 neglected tropical diseases according to the WHO classification (http://www.who.int/neglected_diseases/diseases/en/). The number of people at risk of *O*. *volvulus* infection was estimated to be 113.5 million, and those at risk of LF to be 1.34 billion worldwide [17]. The cornerstone of the fight against onchocerciasis and LF in highly endemic areas in Africa is mass community treatment with ivermectin (Mectizan) alone and in combination with albendazole, respectively.

In loiasis endemic areas where onchocerciasis is mesoendemic or hyperendemic (i.e. prevalence of onchocercal skin nodules in adult males aged ≥20 years higher than 20% and 40% respectively), ivermectin mass treatment is justifiable because the benefit of preventing onchocerciasis associated morbidity outweighs the risk of loiasis-related post-treatment adverse reactions [18].

In LF and loiasis co-endemic areas, the control of LF has not yet or only recently started, partly because of the risk of loiasis-related post-treatment SAEs [19]. This puts the planned elimination of LF at risk [17]. WHO has recently suggested a provisional strategy for interruption of LF transmission in loiasis-endemic areas based on biannual treatment with albendazole complemented with vector control [20, 21].

Studies in Senegal and Mali have shown that 15–17 years of annual or biannual community directed treatment with ivermectin (CDTI) can result in elimination of *O*. *volvulus* transmission [22, 23]. Furthermore, the prevalence of *O*. *volvulus* infection following long term CDTI has been reduced significantly in many other areas [24, 25]. Consequently, the objectives of the African Programme for Onchocerciasis Control were expanded from elimination of onchocerciasis as a public health problem to elimination of *O*. *volvulus* transmission where feasible [26]. This may require treatment of onchocerciasis in hypoendemic areas co-endemic with loiasis. A treatment that can safely reduce LLM below the risk threshold for severe ADRs and SARs for a time sufficiently long to implement ivermectin mass treatment, would be a major contribution to efforts to control and eliminate onchocerciasis and LF. Such a treatment may also be beneficial for patients suffering from loiasis.

Previous trials of the effect of short-term albendazole treatments (1 to 21 days) have shown that albendazole results in a slow reduction of LLM, presumably not due to a microfilaricidal effect but to an effect on the reproductive capacity and/or viability of the macrofilariae. In these studies, the reduction in LLM was either not as extensive as required and/or the study did not include significant number of participants with LLM > 30000 mf/ml [27–30]. The LLM time course in these studies and comparison of the effect of different doses evaluated led to the hypothesis that multiple exposure of the *Loa loa* macrofilariae to albendazole at two months intervals may result in a significant and sustained reduction in LLM.

We report findings from a double-blind, randomized, placebo-controlled trial designed to evaluate whether 2 and/or 6 doses of 800mg albendazole administered at two-months intervals can reduce *Loa loa* microfilaraemia in patients with pre-treatment LLM >15000 mf/ml by at least 50% or even to <8100mf/ml for at least 4 months.

## Methods

### Trial registration

http://www.controlled-trials.com/ISRCTN25831558.

### Study design and intervention

This was a double-blind, randomized, placebo-controlled trial with three parallel treatment arms. Every two months (i.e. at M0, M2, M4, M6, M8, M10), participants received one oral treatment: (1) 800 mg albendazole (6x albendazole arm), (2) 800 mg albendazole at M0 and M2 and matching placebo at M4, M6, M8 and M10 (2x albendazole arm) or (3) matching placebo (placebo arm). LLM was measured during screening, before each treatment, and 14, 18, 21 and 24 months after the first treatment. Before each treatment, all participants had a general medical examination, and women up to 55 years underwent a pregnancy test. Three and seven days after each treatment, participants underwent clinical examination and questioning for any adverse events, to be followed, if clinically indicated, by a laboratory examination. Prior to the 2^nd^ to 6^th^ treatment and at the 14-months follow up, participants were asked about any adverse events since the last evaluation.

### Study area and population

The study was conducted in the Mvila Division in the rain forest of the Southern Region of Cameroon in areas with high loiasis endemicity [1], but <20% prevalence of onchocerciasis (http://www.who.int/apoc/countries/cmr/en/index.html) and thus without CDTI.

Volunteers aged 18–65 years were eligible if they had a LLM >15,000 mf/ml at screening, had no plans to move out of the area over the following 2 years and had given informed consent. Individuals with past or current history of neurological or neuropsychiatric disorders, clinical or laboratory evidence of significant liver and kidney disease, anaemia, intestinal helminth infection, pregnancy, a serious medical condition or any other conditions which should exclude them from the study in the principal investigator’s (JK) opinion, treatment with benzimidazoles during the previous 12 months or with self-reported allergy to benzimidazoles were not eligible.

Participants were identified in a two-step procedure: (1) Screening for *Loa loa* infection: After community mobilization, screening for *Loa loa* infection was performed in the study area from January to March 2007 among all who had given individual written informed consent; (2) Participant selection: Individuals with LLM >15,000 mf/ml at screening and potentially willing to participate in the study, received detailed information about the study, gave informed written consent to study participation and underwent the evaluations to assess their study eligibility. Baseline LLM measurement and first treatment took place between 4 and 12 weeks after screening for loiasis.

### Randomization, blinding and treatment compliance

Eligible individuals were stratified by the LLM obtained during screening: ≤30,000 mf/ml, 30,001 to 50,000 mf/ml and >50,000 mf/ml. Within each stratum, participants were assigned to one of the three treatment arms based on three randomization lists, one for each stratum, prepared by an independent statistician using a random digit table. Lists of eligible participants by stratum were provided to an independent pharmacist not otherwise involved in the study who assigned the treatment on the randomization list for that stratum which corresponded to the position of the participant on the eligible participant list. The pharmacist then prepared treatment packages with the required number of 200 mg albendazole and matching placebo tablets provided by GlaxoSmithKline (GSK).

The treatment packages were provided to the principal investigator (JK) labelled only with participant identifying information which allowed all but the pharmacist to be blinded.

Treatments were taken orally under direct observation by the investigators, 15–30 minutes after a fatty meal (fatty buns with additional ~15g butter). The first treatment occurred in March 2007.

### Laboratory procedures

Calibrated blood smears (CBS) to measure LLM were obtained between 11:00 and 15:00 to account for the diurnal periodicity of *L*. *loa* microfilaria in peripheral blood [31]. For each participant, blood collection was done at the same time of day ± 1 hour throughout the study. Following a finger-prick, 50μl of blood was collected using a 50μl non-heparinized capillary tube and spread on one labelled slide during screening and across two labelled slides during the study for ease and accuracy of counting. The slides were dried at room-temperature, then stained with Giemsa. All *Loa loa* and *Mansonella perstans* microfilariae were counted at 100X magnification. All slides were read by the same blinded biologist throughout the study. A second blinded reading of all slides by that biologist was performed at the end of the study, and the average of the two readings used for data analysis. Differences between the two readings did not exceed 5%.

A Reflotron Plus (Roche) was used to measure blood levels of hemoglobin, alanine aminotransferase (ALAT), aspartate aminotransferase (ASAT), and creatinine. Creatinine clearance was estimated using the Cockroft-Gault formula. Full blood counts were performed using the ABX Pentra-120 flow-cytometer. Pregnancy tests were done using AMS ßHCG urine tests.

### Unblinding and protocol amendment

The protocol initially planned follow-up to 18 months (M18) after the first treatment. Following review of the data after unblinding at M18 by external advisors, a protocol amendment was put in place for LLM measurements 21 and 24 months after the first dose. Participants gave written informed consent for the extended follow-up. All procedures for CBS blood collection and reading of slides remained identical.

### Outcome measures

The primary efficacy variable was the proportion of participants whose LLM was sustainably reduced by ≥50% from the pre-treatment value (value obtained before the first treatment) from any time point after the first dose onward. A sustainable reduction was defined as a reduction at each planned measurement time point over a period of at least 4 months.

Secondary efficacy variables were (1) the proportion of participants whose LLM was sustainably reduced to <8100 mf/ml by strata and by sex, (2) the percent reduction in LLM from pre-treatment at each time point, (3) time course of LLM.

Safety variables were the frequency of adverse events up to four months after the 6^th^ treatment by type, severity, seriousness and relationship to study drug assessed by the investigator while blinded. Severity was graded as mild (event is easily tolerated by the participant, causing minimal discomfort and not interfering with everyday activities), moderate (event is sufficiently discomforting to interfere with everyday activities), severe (event prevents normal everyday activities) or not applicable (events where intensity is meaningless or impossible to determine e.g. blindness). Seriousness was determined based on the serious adverse event definition in the ICH guidelines (any untoward medical occurrence that at any dose results in death, is life-threatening, requires inpatient hospitalization or prolongation of existing hospitalization, results in persistent or significant disability/incapacity or is a congenital anomaly/birth defect and is related to any dose of a medicinal product or the doses normally used in man) [8]. Likelihood of relationship of the adverse event to study drug, i.e. presence of adverse drug reactions (defined as per ICH criteria [8] as 'any noxious and unintended responses to a medicinal product related to any dose or the doses normally used in man'), was assessed based on temporal association with drug administration and biological plausibility taking into account known adverse reactions to albendazole, the participant’s underlying clinical state and known adverse reactions to concomitant treatments.

### Sample size

Assuming that less than 1/1,000,000 placebo treated participants would have a 50% reduction in LLM for at least 4 months and at least 50% of participants receiving 6 albendazole doses would have such a reduction, a sample size of 16 participants per treatment provides ≥90% power to detect the treatment difference at a 2.5% two-sided significance level. The same assumptions were made regarding the effect of 2 albendazole doses. The significance level of 2.5% was chosen based on Bonferoni correction for the two planned comparisons. Assuming attrition of 20% of participants, 20 participants were recruited into each treatment group. 20 participants provide a probability of 0.87 to detect at least one adverse event with a true frequency of 10%.

### Analysis populations

All participants who received at least one dose of study drug were included in the safety analysis and analyzed as randomized.

For the efficacy analyses, participants were analysed as randomized and as part of the stratum they qualified for based on pre-treatment LLM, not the stratum they qualified for based on the screening LLM used for randomization (see Table 2).

**Table 2:** Screening and pre-treatment (baseline) characteristics.

	Placebo (n = 20)	2 albendazole doses (n = 20)	6 albendazole doses (n = 20)	p-value
**Sex**				
Male	9	10	15	0.14
Female	11	10	5	
**Age groups**				
18–39 years old	10	5	5	0.19
40–65 years old	10	15	15	
**LLM) (mf/ml) of subjects at screening (used for determining eligibility and for randomization) and resulting distribution across strata**				
N (%) participants with LLM in stratum 15,000–30,000	11 (55.0)	11 (55.0)	12 (60.0)	
N (%) participants with LLM in stratum 30,001–50,000	5 (25.0)	5 (25.0)	5 (25.0)	0.99*
N (%) participants with LLM in stratum ≥ 50,001	4 (20.0)	4 (20.0)	3 (15.0)	
LLM geometric mean (95% CI)	32,760 (24,374–44,031)	27,916 (22,014–35,402)	29,510 (22,048–39,497)	
LLM arithmetic mean (range)	41,006 (15,900–155,720)	31,549 (15,000–61,400)	37,061 (15,000–152,840)	0.73^t^
LLM median (Interquartile range)	27,130 (20,465–47,155)	28,040 (16,485–44,680)	28,550 (17,030–35,285)	
**LLM) (mf/ml) of subjects before 1**^**st**^ **treatment (baseline) and resulting distribution across strata used for data analysis**				
N (%) participants with LLM in stratum 11,000–30,000	5 (25.0)	10 (50.0)	11 (55.0)	
N (%) participants with LLM in stratum 30,001–50,000	10 (50.0)	4 (20.0)	5 (25.0)	0.20*
N (%) participants with LLM in stratum ≥ 50,001	5 (25.0)	6 (30.0)	4 (20.0)	
LLM geometric mean (95% CI)	44059 (32,733–59,302)	35,206 (25,840–47,967)	33,317 (23,811–46,618)	
LLM arithmetic mean (range)	55,452 (20,860–197,060)	44,905 (15,460–185,920)	44,442 (11,040–169,840)	0.29^t^
LLM median (Interquartile range)	35,690 (29,960–57,055)	30,120 (21,085–64,345)	28,460 (22,975–46,799)	
**Liver enzymes (UI/ml)**				
ASAT/SGOT (geometric mean, 95%CI)	28.0 (22.9–34.3)	35.3 (28.5–43.7)	27.0 (23.7–30.8)	0.12
ALAT/SALAT (geometric mean, 95%CI)	16.0 (12.7–20.3)	19.1 (14.9–24.5)	16.9 (13.1–21.8)	0.43

* Fisher’s exact test.

^t^ Kruskall-Wallis test

All participants with sufficient post-treatment LLM measurements to determine whether or not they had an LLM reduction for ≥ 4 months (i.e. at least two successive measurements over a minimum of 4 months) were included in the efficacy analyses. An intent-to-treat approach was taken with participants being evaluated based on the treatment group they were randomized to, independent of whether they had received the intended number of doses.

### Statistical analysis

#### Screening and baseline characteristics

Screening and baseline characteristics were compared between treatment arms for imbalance. Proportions were compared using Fisher’s exact test, and quantitative variables (ASAT, ALAT, LLM) using the non-parametric Kruskal-Wallis test. Age was transformed into a two-level categorical variable (<40, ≥40 years).

#### Safety analysis

The number and percentage of participants with adverse events was tabulated by type and severity of adverse event, relationship to study drug and treatment group.

#### Efficacy data analysis

The proportions of participants with a sustained, i.e. ≥4 months long reduction in LLM by ≥ 50% (primary efficacy variable) were compared between treatment groups with Fisher's exact test due to the small sample size. The proportion of participants with sustained reduction of LLM to <8100 mf/ml and the percent reduction in LLM from the pre-treatment value by follow-up time point was characterized by descriptive statistics. The longitudinal trend in LLM in each treatment arm up to 11–12 months after the last active dose (i.e. after M2 for the 2x albendazole arm and after M10 for the 6x albendazole arm) and up to the M21 measurement in the placebo group was estimated by fitting a random-effect multiple linear model to the log-transformed LLM as the dependent variable, treatment group and follow-up time as regressors, controlling for age, sex and liver function (ALAT level, in view of the fact that albendazole is converted to the active metabolite albendazole sulfoxide in the liver) [32], and an interaction between follow-up time and treatment. For each treatment group, the resulting regression coefficient was back-transformed to the normal scale by exponentiation and represents the ratio of geometric mean LLM between consecutive data points. The random-effect model takes into account the repeated measurements of LLM over time.

Statistical analyses were performed using Stata 13 (StataCorp LP, College Station, Texas, USA) or using GraphPad Prism version 6.00 and 6.04 for Windows (GraphPad Software, La Jolla, California, USA).

### Ethics statement

The study protocol and protocol amendment received clearance from Cameroon’s National Ethics Committee and from the World Health Organization Ethical Review Committee. The study was granted administrative authorization by the Ministry of Public Health of Cameroon. Study participants gave written informed consent before any study procedures were conducted.

## Results

**Fig 1:**
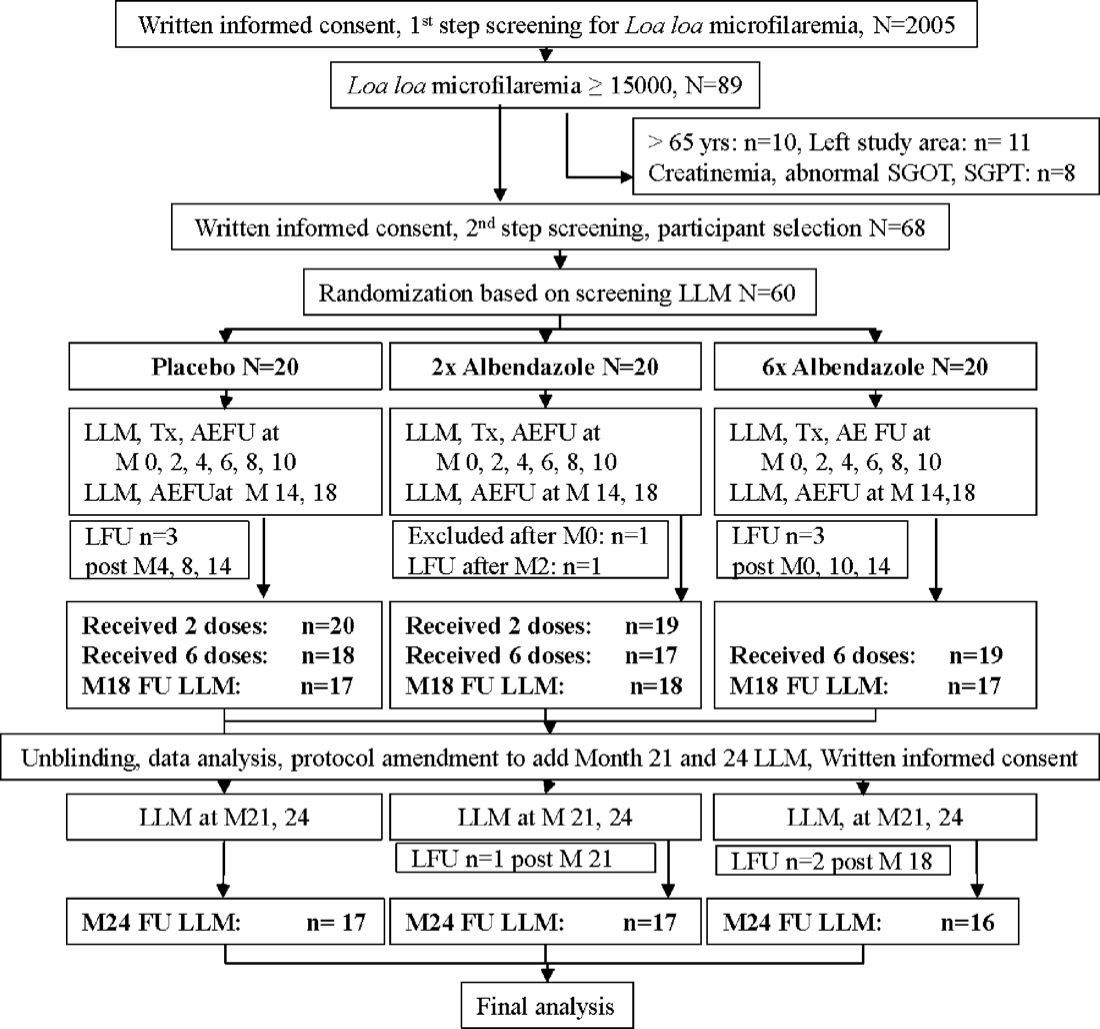
CONSORT flowchart. AE–adverse event, FU–follow up, M–Month, LLM—*Loa loa* microfilaraemia measurement, LFU–Lost to FU, Tx–treatment.

A total of 2005 people from 37 communities were included in the screening for *Loa loa* infection. In villages in which more than 10 participants were examined, between 10% and 49% of those screened were found to be infected. LLM was ≥15,000 mf/ml in 89 people. After screening for inclusion and exclusion criteria, 60 participants were randomized based on the LLM at screening. Screen failure reasons are shown in Fig 1.

### Baseline characteristics

**Fig 2:**
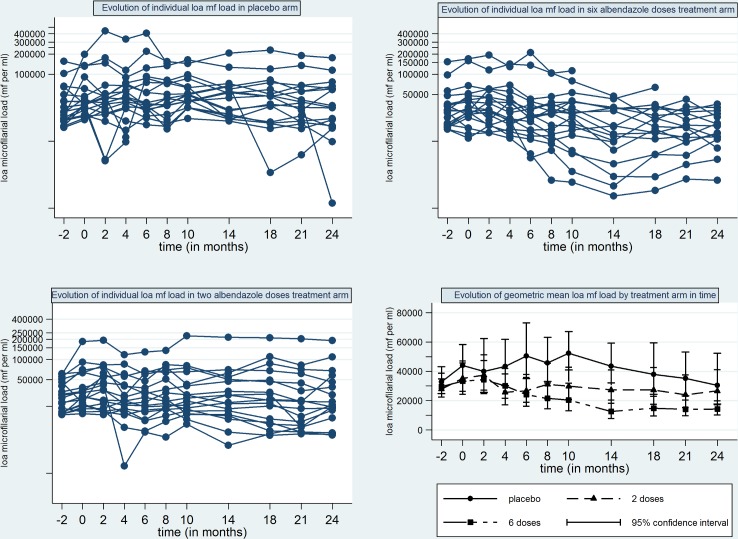
*Loa loa* microfilaraemia in individual participants by treatment group and geometric means of microfilaraemia by treatment group. mf per ml: microfilaria per millilitre of blood.

Table 2 summarizes the LLM data obtained during screening for *Loa loa* infection and at the pre-treatment examination (M0, immediately before the first treatment) as well as other characteristics of the participants. The LLM pre-treatment were in some participants significantly different from the LLM at screening, with pre-treatment LLM ranging from 50% to around 500% of screening LLM. In two participants randomized to the 6x albendazole arm, the LLM dropped from 15000 mf/ml at screening to 11040 mf/ml and 12700 mf/ml, respectively, at the baseline examination. In eight participants randomized to placebo, five participants randomized to 2x albendazole and four participants randomized to 6x albendazole, the LLM measured at baseline was so different from the LLM at screening (Fig 2) that it did not fall within the stratum in which they had been randomized. This resulted in the imbalance in allocation to treatment arms in the different strata when the pre-treatment values are evaluated. There was, however, no statistically significant difference in pre-treatment LLMs between treatment arms (Table 2). As expected, ALAT and ASAT levels were highly correlated (Spearman’s correlation coefficient = 0.77), and only ALAT levels were used in the longitudinal trend analysis.

At baseline, seven participants had detectable levels of *Mansonella perstans* microfilariae including one in the placebo group (440 mf/ml), four in the 2x albendazole group (120 mf/ml, 440 mf/ml, 700 mf/ml, 3820 mf/ml) and two in the 6x albendazole group (200 mf/ml, 1500 mf/ml).

#### Proportion of participants with sustained (≥4 months long) LLM decrease by ≥50% of baseline value and a sustained LLM decrease to <8100 mf/ml

One participant in the placebo arm, two participants in the 2x albendazole arm and one participant in the 6x albendazole arm dropped out of the study before at least 3 consecutive LLM measurements to assess a ≥ 4 months long LLM decrease were obtained. They were thus not evaluable for the primary efficacy analysis or the analysis of the proportion of participants with ≥ 4 months long reduction of LLM to <8100 mf/ml (Fig 1).

Table 3 shows the number and proportion of evaluable participants with sustained LLM decrease by ≥50% from pre-treatment LLM at any time after randomisation and with a sustained LLM of < 8100 mf/ml. The effect of 2x albendazole was not significantly different from that of placebo for either endpoint. In contrast, the proportion of participants with a sustained LLM decrease by ≥50% from the pre-treatment was significantly higher in the 6x albendazole arm than in the placebo arm. There was, however, no significant difference between these two treatment arms in the proportion of participants with a sustained LLM decrease to <8100 mf/ml.

**Table 3:** Proportion of participants with sustained (≥ 4 months) decrease in LLM by ≥ 50% from pre-treatment value and to < 8100 mf/ml.

Endpoint	Placebo	2x Albendazole	6x Albendazole
**Sustained reduction ≥50% from pre-treatment**	**n/N^1^ (%)**	**n/N^1^ (%)**	**n/N^1^ (%)**
Overall**^2^**	2/19 (10.5)	3/18 (16.7)	10/19 (52.6)
Stratum 15 000–30 000 mf/ml	0/5	2/10 (20.0)	6/10 (60.0)
Stratum 30 001–50 000 mf/ml	1/9 (11.1)	1/3 (33.3)	3/5 (60.0)
Stratum >50 000 mf/ml	1/5 (20.0)	0/5 (0)	1/4 (25.0)
**Sustained reduction to <8100mf/ml**	**n/N (%)**	**n/N (%)**	**n/N (%)**
Overall**^3^**	0/19	2/18 (11.1)	4/19 (21.1)
Stratum 15 000–30 000 mf/ml	0/5	1/10 (10.0)	4/12 (33.3)
Stratum 30 001–50 000 mf/ml	0/9	1/3 (33.3%)	0/5
Stratum >50 000 mf/ml	0/5	0/5	0/4

^**1**^ N = number of participants evaluable, i.e. with ≥ three successive LLM measurements following the 1^st^ treatment allowing to assess whether the LLM decrease lasted ≥ 4 months.

^**2**^ Fisher's exact test: Placebo vs. 2x Albendazole p = 0.66, Placebo vs. 6x Albendazole p = 0.01.

^**3**^ Fisher’s exact test: Placebo vs. 2x Albendazole p = 0.23, Placebo vs. 6x Albendazole p = 0.11.

Table 4 shows the treatment, the pre-treatment LLM, the first and last month at which a sustained decrease of LLM by ≥50 from pre-treatment, to <8100 mf/ml as well as to <30000 mf/ml were measured and the number of albendazole doses received prior to the start of the sustained LLM decrease for each participant with a sustained LLM decrease by ≥50% from pre-treatment. The variability of the first month of any of the sustained LLM reductions and the number of albendazole doses taken at that time suggests that factors other than an effect of albendazole impact the LLM.

**Table 4:** Pre-treatment LLM, month of start and end of sustained (≥ 4 months) decrease in LLM by ≥ 50% from pre-treatment LLM, to < 8100 mf/ml or < 30000 mf/ml by treatment arm for participants with a sustained decrease by ≥ 50%.

Treatment, participant number	Pre- treatment LLM (sex)	Start to end^1^ of sustained reduction by ≥ 50% (doses^2^)	Start to end^1^ of sustained reduction to ≤ 8100 mf/ml (doses^2^)	Start to end^1^ of sustained reduction to <30000 mf/ml (doses^2^)
Plac—15	46420 (F)	M18-M24	NA	M18-M24
Plac—17	90940 (M)	M14-M24	NA	NA
2x Alb—2	16600 (M)	M14-M24 (2)	M18-M24 (2)	NA
2x Alb—10	28120 (F)	M14-M24 (2)	NA	NA
2x Alb—13	34120 (M)	M4-M24 (2)	M14-M18 (2)	M4-M24 (2)
6x Alb—1	11040 (F)	M14-M24 (2)	M10-M24 (5)	NA
6x Alb—3	15500 (M)	M6-M24 (3)	M6-M24 (3)	NA
6x Alb—5	22460 (F)	M6-M24 (3)	M10-M21 (4)	NA
6x Alb—6	24520 (M)	M14-M21 (6)	M6-M18 (3)	NA
6x Alb—7	25520 (M)	M6-M21 (3)	NA	NA
6x Alb—8	27240 (M)	M18-M24 (6)	NA	NA
6x Alb—12	32040 (M)	M14-M24 (6)	NA	M2-M24 (1)
6x Alb -14	41180 (M)	M18-M24 (6)	NA	M10-M24 (5)
6x Alb—15	42980 (M)	M14-M24 (6)	NA	M10-M24 (5)
6x Alb—19	158400 (M)	M10-M24 (5)	NA	NA

^**1**^ Start and end are provided as the month M of the first and last measurement of the sustained LLM reduction.

^**2**^ Doses: number of albendazole doses received before the start of the sustained reduction. The first dose was given at M0

NA: not achieved or not applicable (for reduction to <30000 mf/ml for participants with < 30000mf/ml pre-treatment LLM)

#### Reduction from pre-treatment value in *Loa loa* microfilaraemia by time point

**Fig 3:**
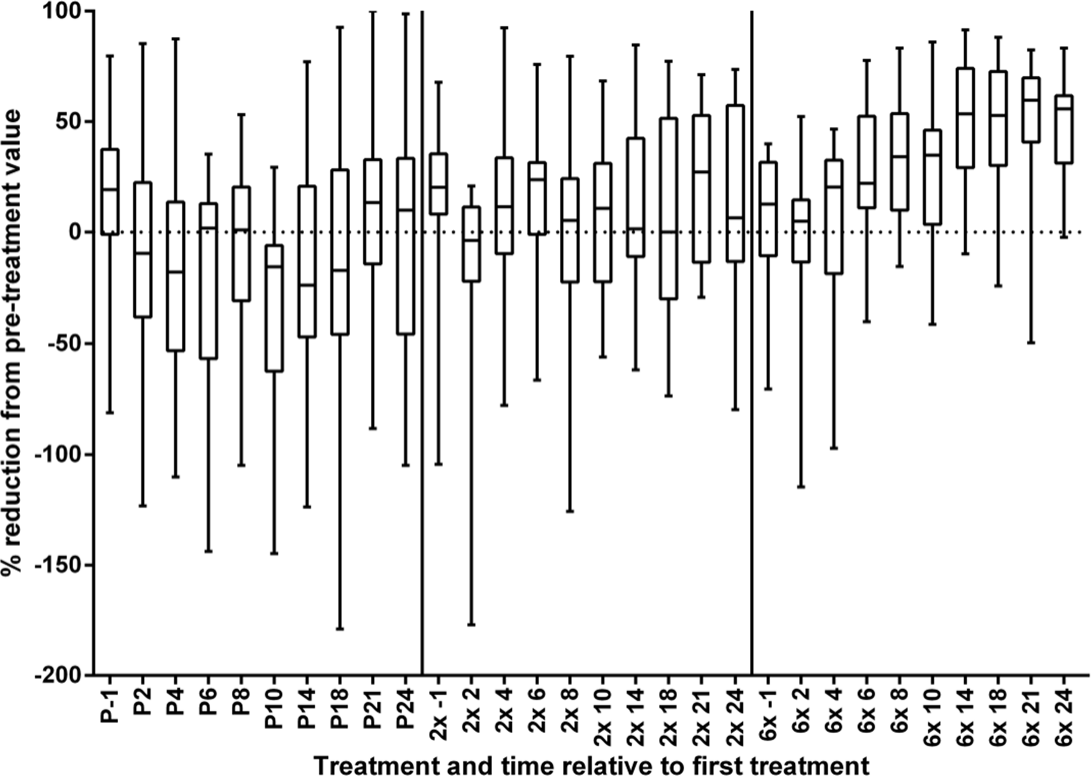
Minimum, 25%, 50th, 75th percentile and maximum of the % reduction from pre-treatment values. P placebo, 2x 2 doses albendazole, 6x 6 doses albendazole, -1 value obtained during screening (4–12 weeks prior to baseline measurement and first treatment), 2, 4, 6, 8, 10, 14, 18, 21, 24 Months after the first treatment at which LLM was measured.

Fig 3 shows for each treatment arm and time point following the first treatment the maximum, 75^th^ percentile, median, 25^th^ percentile and minimum of the percent reduction of LLM from the value measured before the first treatment. Furthermore, Fig 3 includes the difference between screening and pre-treatment LLM calculated as reduction in percent from pre-treatment value (indicated as time point '-1' relative to treatment). It was included in the figure to facilitate comparison of the post-treatment LLM reductions with the significant LLM changes which occurred between screening and pre-treatment measurement. The large variations in the change from pre-treatment value in both directions in each treatment group reflect the significant intra- and inter-participant variation in LLM evident in Fig 2. Considering the low proportion of participants receiving 2x or 6x albendazole with sustained LLM reductions reported in Table 3, descriptive analysis of the reductions by strata and sex was not performed.

#### Longitudinal trends in LLM from baseline to 11–12 months after completion of active treatment

The results of the linear trend analysis (Table 5) confirm the conclusions from the review of the LLM time courses (Fig 2) and the reduction from baseline (Fig 3). There was no significant LLM change in the placebo or 2x albendazole group. The significant inter-participant variability in response to 6x albendazole (Fig 2, Fig 3, Table 5) is reflected in the very wide 95% confidence intervals of the regression coefficients.

**Table 5:** Longitudinal trend in *Loa loa* microfilaraemia from baseline up to 11–12 months after completion of each regimen.

Treatment arm	Crude regression coefficient (95% CI) ^2^	p-value	Adjusted^1^ regression coefficient (95% CI) ^2^	p-value
Placebo	0.99 (0.97–1.00)	0.21	0.99 (0.97–1.01)	0.21
2x albendazole	0.71 (0.46–1.10)	0.13	0.69 (0.44–1.09)	0.11
6x albendazole	0.69 (0.43–1.09)	0.11	0.59 (0.37–0.92)	0.02

The 2-doses regimen was completed at M2, and data from baseline up to month 14 included in the analysis; the 6-doses regimen was completed at M10, and data from baseline to month 21 was included in the analysis. The analysis of the placebo group included the data up to month.

^1^ Adjustment for age, sex and baseline liver function (ALAT)

^2^ Regression coefficients represent the ratio between geometric means of consecutive cross-sectional measurement within each group, hence describing an exponential progression.

### Adverse events

Up to 2 months after the last albendazole or placebo dose administered, 15, 13 and 15 participants were found to have a total of 46, 48 and 45 adverse events, respectively, in the placebo, 2x albendazole and 6x albendazole arm. None was regarded as study drug related. Across all participants, the most frequently reported AEs were different types of pain (e.g. arthralgia, myalgia, back pain, pain in extremities) and malaria.

The AEs were of mild or moderate intensity except for three severe adverse events which also met the criteria for SAEs (see Table 1). Upon unblinding, the participants with SAEs were found to have been in the placebo group. One participant sustained a severe chest trauma in a fight two days before the sixth treatment. One participant developed severe malaria and typhoid fever one week after the first treatment and was excluded from further treatment. Another participant, a 47 year old man, died 6 weeks after the 5^th^ treatment; his death occurred at home and was preceded by a short illness including fever, cough, seizure and coma. The post-mortem was not able to establish the cause of death. The *Loa loa* microfilaraemia was 40,000 mf/ml at screening, 197,000 mf/ml at baseline and 440,080 mf/ml, 332,060 mf/ml, 410,200 mf/ml and 144,360 mf/ml two months after the first, second, third and fourth placebo dose, respectively. The pathology found minor brain haemorrhages, suggesting that *Loa loa* infection could have been a contributing factor.

### Observations on *M*. *perstans* microfilariae levels

**Fig 4:**
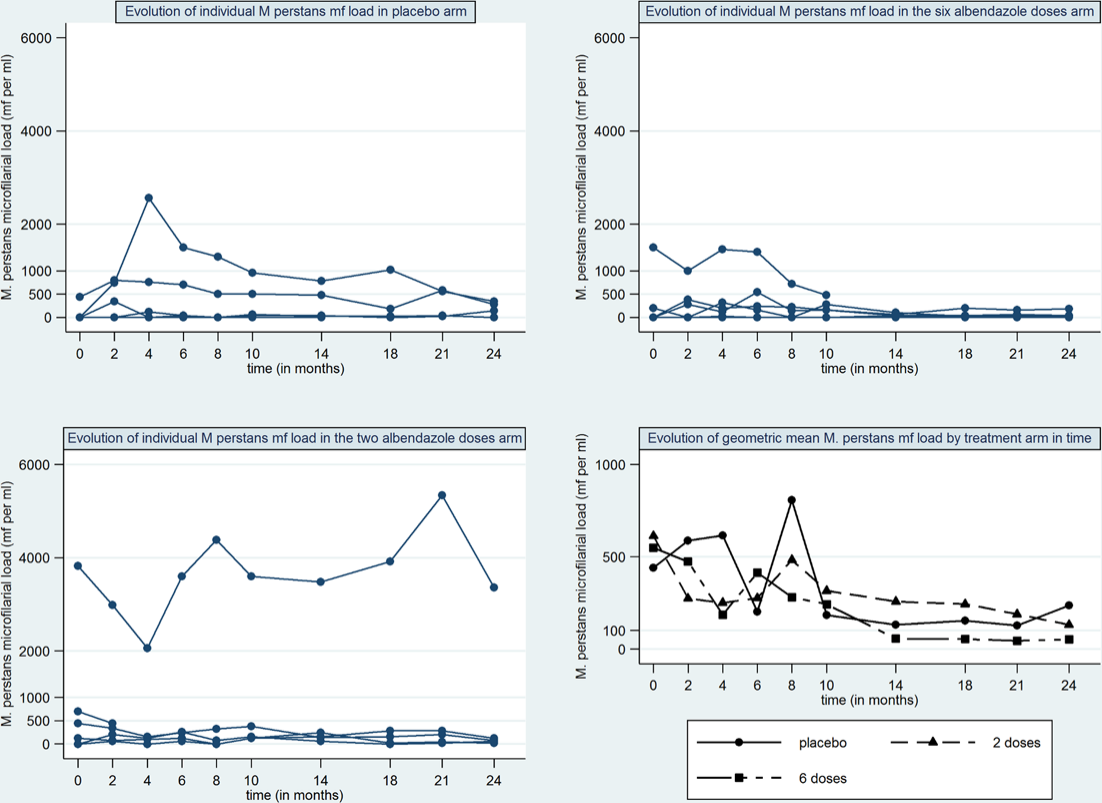
*M*. *perstans* microfilariae levels in participants in whom *M*. *perstans* was detectable at least once during the study by treatment group. mf per ml: microfilaria per millilitre of blood.

During the 24 month follow up period, 11 participants who did not have detectable levels of *M*. *perstans* at baseline, had detectable levels at least once. In some participants, *M*. *perstans* levels varied significantly over time without any indication of an effect of 2x or 6x albendazole. The *M*. *perstans* microfilariae levels in all participants in whom detectable levels were detected at least once are shown in Fig 4.

## Discussion

As anticipated based on prior knowledge of albendazole [27–30, 33–36], no mild, moderate or severe adverse drug reactions were recorded.

This study evaluated whether a 2 dose and/or a 6 dose albendazole treatment regimen result in a ≥50% reduction in LLM from pre-treatment for at least 4 months. Depending on the extent and duration of LLM reduction, the regimen could be considered for community wide treatment in *Loa loa* co-endemic areas before ivermectin or ivermectin-albendazole mass treatment to reduce the risk of severe ADRs or SARs or for further improvement of the regimen.

The results show that only the 6 dose regimen had a LLM reducing effect. The time course of LLM reduction, and the absence of adverse drug reactions known to occur upon treatment of individuals with high LLM with microfilaricidal drugs support the conclusions from prior studies [28–30] that the LLM reducing effect of albendazole is likely due to microfilariae dying as they reach the end of their life span. They are not being replaced because albendazole binding to β-tubulin disrupts microtubule structure, function and formation which results in macrofilariae starvation and inhibition of reproduction [37, 38].

Around 50% of participants in the 6x albendazole arm experienced a sustained LLM decrease by ≥ 50%. None of the 9 participants with LLM >30000 mf/ml before treatment had a sustained LLM decrease to <8100 mf/ml. The LLM lowering effect of the 6x albendazole regimen is therefore insufficient to significantly reduce the population at highest risk of severe ADRs or SARs upon ivermectin mass treatment. This study thus adds to the body of data showing insufficient efficacy of different regimens of albendazole for reducing LLM. In contrast to our study, these studies included participants with pre-treatment LLM <8100 mf/ml, no or an unspecified number of participants with LLM > 30000 mf/ml and the results were presented only via summary statistics across all participants [27–30].

The cost-benefit of further efforts to improve an albendazole based treatment regimen, e.g. through sustained release formulation technology, for LLM reduction needs to be carefully considered. These considerations need to take into account the dose- and time-dependent pharmacokinetics of albendazole, including the inter-subject variability in albendazole bioavailability and conversion to the active metabolite albendazole sulfoxide, the fact that albendazole induces its own disposal during long term treatment resulting in decreasing levels of albendazole sulfoxide [39–41], the potential toxicity associated with long term exposure (http://www.accessdata.fda.gov/drugsatfda_docs/label/2015/020666s009lbl.pdf) and the time and cost to develop an affordable, safe and efficacious dose. These considerations also need to include the alternative drugs and approaches in development. An oral flubendazole formulation is now being evaluated for clinical development for onchocerciasis [42]. Prior clinical data suggest that flubendazole does not have a microfilaricidal effect, but leads to a slow reduction in microfilariae levels through an effect on the *O*. *volvulus* macrofilariae [43]. Large scale efforts to discover novel antibiotics targeting the *Wolbachia* endosymbionts in the filariae that cause onchocerciasis and lymphatic filariasis are under way [44]. Doxycycline treatment of *O*. *volvulus* infected individuals has provided proof-of-concept for the effect of antibiotics on the reproductive activity and viability of the macrofilariae without microfilaricidal activity [45, 46]. One study of doxycycline in *O*. *volvulus* infected people included 22 people with a pre-treatment LLM of < 8000 mf/ml. No adverse events of the type and severity observed after ivermectin treatment of people with high LLM were reported[47].

Alternate approaches to onchocerciasis and lymphatic filariasis control in *Loa loa* co-endemic areas are under evaluation. This includes development of diagnostics for high levels of infection with *Loa loa* to identify individuals at risk for severe ADRs and/or SARs to ivermectin [48]. If the ongoing field testing is successful, *Loa loa* infected individuals at risk of ADRs/SARs and co-infected with *O*. *volvulus*, could be treated with regimens of antibiotics already shown to be effective against *O*. *volvulus*. The implementation of this approach, including the 'cut-off' for exclusion from ivermectin treatment and the time between LLM measurements and treatment, needs to take into account the substantial intra-individual LLM variability observed in the absence of treatment in this study (Table 2, Fig 2, Fig 3). Research on LLM variability within shorter intervals than the 1–4 months in our study may be needed to inform the maximum time frame between LLM measurement and safe ivermectin treatment.

The level of variability we observed in the absence as well as during and after treatment has to our knowledge not previously been reported. It needs to be taken into account during review of the other studies which evaluated the effect of albendazole regimens on LLM based on summary statistics [27–30]. Analysis of the data from this study via geometric mean LLM (Fig 2), shows a progressive LLM decrease in the 6x albendazole arm from M2-M14 to around 50% of pretreatment levels. Only the review of the individual participant data (Fig 2, Table 3) showed that this mean decrease was driven by only a few individuals and that start time relative to treatment and the duration of LLM decrease differed between individuals (Table 4). Any treatment to ensure safe ivermectin mass treatment has, however, to reduce LLM below the level of risk in all to be treated with ivermectin and the start time relative to treatment and the duration of the LLM decrease below the risk level needs to reliable. Consequently, LLM variability needs to be taken into account in the design, analysis and reporting of all future studies on the efficacy and safety of drugs or strategies for addressing loiasis as an obstacle for onchocerciasis and lymphatic filariasis control and elimination and as a neglected disease that can negatively impact people's health, well-being and health care costs.

## Supporting Information

S1 Checklist Consort checklist. (DOCX)

S1 Database *Loa loa* Alben Trial data. Variables: Loa Mx- *Loa loa* mf/50 μl at month x, Perstans Mx- *Mansonella perstans* mf/50 μlat month x.
(XLSX)

S1 Protocol Protocol of the trial with amendment. (PDF)
