# Supplementary material for: Effect of Two or Six Doses 800 mg of Albendazole Every Two Months on Loa loa Microfilaraemia: A Double Blind, Randomized, Placebo-Controlled Trial
Source: PLoS Negl Trop Dis. 2016 Mar 11;10(3):e0004492. doi: 10.1371/journal.pntd.0004492 (PMC4788450; doi:10.1371/journal.pntd.0004492)
Supplement: S1 Protocol — (PDF) [file pntd.0004492.s003.pdf]

Efficacy of albendazole in decreasing *Loa loa* microfilaraemia

**Protocol title**	**Safety and efficacy of albendazole for treatment of Loa loa infection**
**Product**	**Albendazole**
**Phase**	**Non-regulatory, post-registration study**
**Date of Protocol**	**March 2006**
**Protocol version**	**Version 1.0**
**Design**	**Randomized, Double blind, placebo controlled**
**Study site**	**Coalition des ONGD Internationales contre l'onchocercose (Loiasis Technical Advisor offices)**
**Principal Investigator**	**Joseph Kamgno, MD, MPH, PhD.**
	**Coalition des ONGD Internationales contre l'onchocercose**
	**BP 4794 Yaoundé, Cameroun**
	**Phone: +(237) 778 97 36**
	**Fax: +(237) 221 55 67**
	**e-mail: kamgno@pasteur-yaounde.org, djoekam@hotmail.com**
**Sub-investigator**	**Mathurin Cyrille Téjiokem**
	**Centre Pasteur du Cameroun**
	**BP 1274 Yaounde, Cameroun**
	**Phone: +(237) 764 52 58**
	**Fax: +(237) 223 15 64**
**Laboratory**	**Coalition des ONGD Internationales contre l'onchocercose (Loiasis Technical Advisor offices)**
	**BP 4794 Yaoundé, Cameroun**
**Data management and analysis**	**Coalition des ONGD Internationales contre l'onchocercose (Loiasis Technical Advisor offices)**<br>**BP 4794 Yaoundé, Cameroun**
**Product Manager**	**Annette Kuesel, PhD**
	**TDR/PDE**
	**World Health Organisation, 20 Avenue Appia, CH-1211 Geneva 27, Switzerland Tel: +41 22 791 3818**
	**Fax: +41 22 791 4774 Email: Kuesela@who.int**
**Sponsor**	**UNICEF/UNDP/World Bank/WHO Special Programme for Research and Training in Tropical diseases, , 20, Avenue Appia, CH-1211 Geneva 27, Switzerland**

Signature Page

The protocol and the supporting documents contain all the necessary details for carrying out the study.

I will provide copies of the protocol and access to all information furnished by the sponsor to our sub-investigator and other members of the study team. I will discuss this material with them to ensure that they are fully informed about the study and their responsibilities within the study.

I agree to ensure that this study is conducted as described, and in full accordance with Good Clinical and Laboratory Practices, the guidelines in the TDR SOP for clinical investigators (TDR SOP.01.1) and all applicable rules and regulations in Cameroon.

Principal Investigator
J. Kamgno, MD

Investigator's Signature

16/05/2006
Date: DD/MM/YY

APPROVED FOR SUBMISSION TO ERC

TDR Clinical Coordinator Name
Juntra Karbwang, MD PhD

Clinical Coordinator Signature

Date: DD/MM/YY

TDR Product Manager's Name
Annette C. Kuesel, PhD

Product Manager's Signature

Date: DD/MM/YY

Signature Page

The protocol and the supporting documents contain all the necessary details for carrying out the study.

I will provide copies of the protocol and access to all information furnished by the sponsor to our sub-investigator and other members of the study team. I will discuss this material with them to ensure that they are fully informed about the study and their responsibilities within the study.

I agree to ensure that this study is conducted as described, and in full accordance with Good Clinical and Laboratory Practices, the guidelines in the TDR SOP for clinical investigators (TDR SOP.01.1) and all applicable rules and regulations in Cameroon.

Principal Investigator<br>J. Kamgno, MD	Investigator's Signature	Date: DD/MM/YY

APPROVED FOR SUBMISSION TO ERC

TDR Clinical Coordinator Name<br>Juntra Karbwang, MD PhD	[signature]<br>Clinical Coordinator Signature	9 May 06<br>Date: DD/MM/YY
TDR Product Manager's Name<br>Annette C. Kuesel, PhD	[signature]<br>Product Manager's Signature	8 May 06<br>Date: DD/MM/YY

Ethics committees who reviewed this protocol

IEC:	National Ethics Committee of Cameroon
Address:	BP 1937 Yaoundé, Cameroon Tél. 220 90 75

IEC:	World Health Organization, Research Ethics Review Committee
Affiliation:	World Health Organization
Address:	20 Avenue Appia, CH 1211 Geneva 27, Switzerland

Table of contents

Signature Page 2
Ethics committees who reviewed this protocol 3
Table of contents 4
Table of Tables 7
Table of Figures 7
Abbreviations 7
1 TABULAR OVERVIEW 9
2 STUDY FLOW CHART 12
3 RATIONALE AND BACKGROUND 13
4 STUDY GOAL, OBJECTIVES AND EXPECTED OUTCOMES 20
5 STUDY DESIGN 20
5.1 Overview 20
5.2 Primary endpoint 22
5.3 Secondary endpoints 22
6 METHODOLOGY 23
6.1 Selection of study population and acquisition of baseline data 23
6.1.1 Inclusion criteria 23
6.1.2 Exclusion criteria 24
6.1.3 Conduct of screening 24
6.1.3.1 Village information about study and village focal point 24
6.1.3.2 Preliminary survey 24
6.1.3.3 Definitive subject qualification for the study 25
6.1.3.4 Definitive subject selection 25
6.1.3.5 Baseline LLM in subjects selected for study participation 26
6.1.3.6 Subject information on screening results 26
6.1.4 Subject identification and screening log 26
6.1.5 Screen failures 26
6.1.6 Subject withdrawal 27
6.1.6.1 Withdrawal from the study 27
6.1.6.2 Withdrawal from study treatment 27
6.1.6.3 Reasons for withdrawal 27
6.1.7 Description of methods used during screening and baseline data acquisition 28
6.1.7.1 Parasitological examination 28

6.1.7.2 Clinical examination 28

6.1.7.3 Laboratory evaluations 28

6.2 SUBJECT TREATMENT 28

6.2.1 Study drugs and treatment regimen 28

6.2.2 Choice of control treatment 29

6.2.3 Determination of compliance 29

6.2.4 Measures to minimize bias 29

6.2.4.1 Blinding during treatment and unblinding in case of emergencies 29

6.2.4.2 Randomization 30

6.2.5 Study drug preparation 30

6.2.6 Study drug administration and recording 30

6.2.7 Accountability for study drug 31

6.2.8 Prohibited and permitted prior treatments 31

6.2.9 Prohibited concomitant treatment 31

7 ASSESSMENT OF EFFICACY 32

7.1 Efficacy Variables 32

7.1.1 Primary 32

7.1.2 Secondary 32

7.2 Method and timing of measurement of efficacy parameter 32

8 ASSESSMENT OF SAFETY 33

8.1 Safety Variables 33

8.2 Methods and timing for assessing adverse events 33

8.3 Adverse event definitions 34

8.3.1 Adverse event 34

8.3.2 Treatment emergent adverse events 34

8.3.3 Serious adverse event 34

8.4 Assessment of severity of adverse events 35

8.5 Assessment of relationship of adverse event to study drug 35

8.6 Reporting of serious adverse events and pregnancies to GSK, TDR and National regulatory authorities 36

8.6.1 Serious adverse events 36

8.6.2 Pregnancies 36

9 DATA ANALYSIS 36

9.1 Sample Size Justification 36

9.2 Statistical Methods 36
9.2.1 Efficacy analysis 36
9.2.2 Safety analysis 37
9.2.3 Analysis populations 37
9.2.4 Interim Analysis 37
9.2.5 Reasons for discontinuation of treatment/study 37
10 PROBLEMS ANTICIPATED 37
11 ETHICS 38
11.1 Informed consent 38
11.2 Gender related issues 38
11.3 Potential risks to subjects 38
11.4 Potential benefits to subjects 38
11.5 Potential benefits to communities 39
11.6 Inclusion of a placebo arm 39
11.7 Data safety monitoring 39
11.8 Information of the communities of the study results 39
11.9 Subject Compensation 39
11.10 Insurance 39
12 OBLIGATIONS OF THE INVESTIGATOR 39
12.1 Ethics Committee Approval 39
12.2 Pre study Documentation 40
12.3 Study management 40
12.4 Informed Consent 40
12.5 Serious Adverse Event Reporting 40
12.6 Source Records 40
12.7 Protocol Amendment 41
12.8 Quality Assurance 41
12.9 Interim Report 41
12.10 Final Study Report 41
12.11 Record retention 42
12.12 Change in Investigator(s) 42
12.13 Confidentiality 42
13 TERMINATION OF THE STUDY 42
13.1 Termination by the Sponsor 42
13.2 Termination by the Investigator 42

14 QUALITY ASSURANCE 42

15 PUBLICATIONS 42

16 REFERENCES 43

17 ATTACHMENTS 45

Table of Tables

Table 1 Overview of severe or serious neurological events reported in the literature...... 14

Table 2 Overview of effect of albendazole treatment on *Loa loa* microfilaremia............. 19

Table of Figures

Figure 1 *Loa loa* microfilaremia counts and diurnal change in *Loa loa* infected subjects treated with ivermectin in Cameroon.. 22

Abbreviations

**Abbreviation**	**Term**
AE	Adverse event
ALT	Alanine aminotransferase (SGPT)
AST	Aspartate aminotransferase (SGOT)
BID	Twice a day
BUN	Blood urea nitrogen
CBC	Complete blood count
CBS	Calibrated blood smear
CDTI	Community directed treatment with ivermectin
CE	Clinical Examination
CRF	Case report form
CV	Curriculum vitae
DEC	Diethyl-carbamazine
EC	Ethics committee
ERC	WHO Ethics Review Committee
GCP	Good clinical practice
GSK	Glaxo-SmithKline
HCG	Human choriogonadotropin
ICH	International Conferences on Harmonization
IEC	Independent ethics committee
LF	Lymphatic filariasis
LLM	*Loa loa* microfilaremia
MDA	Mass Drug Administration

Abbreviation	Term
MEC	Mectizan Expert Committee
mf	Microfilariae
OTC	Over the counter
PDE	Product Development and Evaluation
PELF	Programme to Eliminate Lymphatic Filariasis
PI	Principal investigator
PLERM	Probable or possible *Loa loa* encephalopathy related to treatment with Mectizan.<br>*Loa loa* encephalopathy related to treatment with Mectizan: Encephalopathy (without seizures, usually with fever) in a person previously healthy and without another underlying cause for emcephalopathy, onset of progressive CNS symptoms and signs within 7 days of treatment with ivermectin, illness progressing to coma without remission.<br>Assessment of probability of relationship to Mectizan treatment:<br>Probable if peripheral blood *L.loa* >1000 mf/ml pretreatment or >1000 mf/ml within 1 month post treatment or >2700 mf/ml within 6 months of treatment, and/or *L.loa* microfilarae in the CSF.<br>Possible if semi-quantiative or non-quantitative positive (i.e. +, +, +++) *L.loa* microfilariae in pheripheral blood within 1 month post treatment.
PT	Pregnancy test
QD	Every day
qnh	Every n hours
SAE	Serious adverse event
TID	Three time per day
TDR	UNICEF/UNDP/World Bank/WHO Special Program for Research and Training in Tropical Diseases
ULN	Upper limit of normal
WBC	White blood cell
WHO	World Health Organization
WNL	Within normal limits

1 TABULAR OVERVIEW

**Study Title**	Efficacy of albendazole in decreasing *Loa loa* microfilaraemia
**Clinical Phase**	Non-regulatory study, Phase 2 (proof of concept)
**Study Rationale**	Loiasis is a filarial infection endemic in the rain forest of Subsaharan Africa. In most cases, the infection is benign and does not result in disability or severe clinical symptoms. It is not known to what extent infection is associated with fatigue, recurrent fever or perhaps arthritic pains or to what extent the eosinophilia seen in some infected subjects may contribute to endomyocardial fibrosis.<br>Diethylcarbamazine is effective against *Loa loa* macrofilaria, but can cause serious complications such as meningoencephalitis, which is sometimes fatal.<br>Ivermectin is very effective against microfilaria but can also be associated with serious and/or severe adverse events in subjects with high *Loa loa* microfilaria load (for more details see further below). Available data to date suggest that the risk of adverse events increases with *L.loa* microfilaria load. Subjects with loa parasitemia >8 100 mf/ml have an increased risk of developing reactions that result in functional impairment for at least several days compared to subjects with no detectable loa loa parasitemia (Gardon et al. 1997). Serious neurological reactions including encephalopathies have been described in subjects with measured or estimated pre-ivermectin treatment loa parasitemia of >30 000 mf/ml (Ducorps et al. 1995, Gardon et al. 1997, MEC 2002, MEC 2003). Most of the patients with serious neurological reactions recovered without any sequelae but some patients died or recovered with neurological and/or physical sequelae. Thus, there is no safe treatment to treat or cure *Loa loa* infection.<br>Ivermectin mass treatments of populations via community directed treatment (CDTI) in areas meso- or hyper-endemic for onchocerciasis is the cornerstone of onchocerciasis control programmes. Similarly, yearly mass treatment with albendazole and ivermectin is the cornerstone of the efforts to eliminate lymphatic filariasis (LF) in Africa in areas where LF and onchocerciasis are coendemic. Because of the risk of serious and/or severe adverse events associated with ivermectin treatment in subjects with loiasis, ivermectin mass treatment for onchocerciasis or LF is problematic in areas co-endemic for loiasis:<br>In the absence of mass treatment suitable methods to identify subjects with high loa loa microfilaremia (LLM), the only way to eliminate the risk of the severe and/or serious adverse events during onchocerciasis control is to exclude areas with high prevalence and intensity of *L. loa* infection from standard ivermectin mass treatments. In areas where onchocerciasis and loiasis are co-endemic, ivermectin treatment is initiated only once appropriate risk management measures have been put in place (Mectizan Expert Committee and The Technical Consultative Committee, 2004). LF control programs are not being expanded into areas co-endemic with loiasis.<br>Thus, a safe treatment reducing LLM would not only benefit *Loa loa* infected subjects by reducing loiasis related signs and symptoms and the probability of severe long term sequelae of the infection, but if sufficiently effective to reduce LLM below the level associated with risk of serious and/or severe adverse events prior to treatment with ivermectin (alone or in combination with albendazole) could prevent the occurrence of these

	AEs in *Loa loa* infected subjects with onchocerciasis and/or lymphatic filariasis.<br>A safe treatment would reduce LLM not by killing the microfilaria but rather by preventing the generation of new microfilariae so that LLM is reduced slowly as the microfilariae reach the end of their natural life span.<br>Previous experience with albendazole suggests that albendazole does not kill *Loa loa* microfilariae, but rather exerts a macrofilaricidal or macrofilaria sterilizing effect. The short term albendazole treatment regimens evaluated to date have not resulted in a sufficient and sustained reduction of *Loa loa* microfilaremia. The time course of LLM following the treatment regimens evaluated to date, suggests that multiple administration of this drug every two months several times may achieve a significant progressive slow decrease of *Loa* parasitemia.<br>This trial will evaluate the effect of 800 mg albendazole administered twice or 6 times every two months on LLM.
**Trial Design**	Randomized, double-blind, placebo-controlled, field trial
**Approximate Duration of Subject Participation**	Up to 2 months between initiation of screening and treatment<br>18 months between first treatment and last follow up.
**Approximate Duration of Study**	21 months, including<br>2 months between initiation of screening and enrolment<br>19 months of treatment and follow up period across all subjects
**Study Objective(s)**	Primary objective<br>Evaluate the efficacy of two different albendazole treatment regimens in sustainably reducing *L. loa* microfilarial (mf) load in subjects with pretreatment levels >15000 mf/ml<br>Secondary objective<br>Assess the safety of these treatment regimens in the study subjects
**Diagnosis and Main Criteria for Inclusion**	18-65 year old males and non-pregnant females with parasitologically confirmed *Loa loa* parasitaemia of >15000 microfilaria/ml who are otherwise healthy
**Main Criteria for Exclusion**	Subjects who meet ANY of the following criteria will be excluded from the study<br>- Treatment with a benzimidazole during the last 12 months<br>- Self-reported allergy to benzimidazoles.<br>- Pregnancy<br>- Clinical signs and symptoms and laboratory evidence of intestinal helminths<br>- Any serious underlying medical condition<br>- Past or current history of neurological or neuropsychiatric disorders<br>- Clinical and/or laboratory evidence of significant liver disease, kidney disease or anaemia or any other condition that in the investigator's judgment should exclude the subject from the study.
**Number of Subjects**	60 subjects, including 20 treated with placebo, and 20 each treated with one of the two albendazole dosing regimens
**Number of Study Centers**	Single center study
**Concomitant**	None

**Treatment**	
**Study Treatment**	Albendazole tablets (200 mg) and matching placebo<br>Administration: 800 mg albendazole orally every two months 0 (group 1, placebo), 2 (group 2, treatment regimen 1) or 6 times (group 3, treatment regimen 2).<br>Albendazol has a well-established excellent safety profile which allows it to be administered without medical supervision not only to infected, but also to uninfected subjects during the mass treatment of populations living in areas endemic for LF.
**Efficacy Evaluation**	*L. loa* microfilaremia determined via calibrated blood smears (CBS) pre-treatment and 2, 4, 6, 8, 10, 14 and 18 months after the first treatment. Blood samples for CBS will be obtained at around the same time between 11:00 and 15:00 o'clock to account for the diurnal variation of *L.loa* counts in the blood.
**Safety Evaluation**	On the day of treatment as well as 3 and 7 days after each treatment subjects will be evaluated clinically and for self-reported adverse events. On the day of the next treatment, subjects will be asked for adverse events experienced since day 7 after the previous treatment. Laboratory tests will be performed at screening and if indicated based on the results of the clinical investigation or based on subject reported AEs.
**Statistical Analysis**	The proportion of subjects with a sustainable (≥ 4 months) reduction of LLM by ≥50% of the baseline value by treatment group across all subjects and by initial microfilaria load, will be compared with the Chi-squared test.<br>Comparison of *L. loa* mf/ml between treatment groups at each time point with non-parametric tests due to non-normal distribution of mf loads. General linear model for analysis of the evolution of microfilaria load in time in each treatment group.
**Rationale for Number of Subjects**	A positive response in a subject is defined as a drop in LLM from baseline values by ≥50% lasting at least 4 months. Assuming that in the population less than $1/10^6$ subjects will have a positive response if randomized to the placebo group and at least 50% of the subjects will have a positive response if randomized to the 6 dose albendazole treatment regimen, a sample size of 16 subjects in each treatment arm will provide at least 90% power to detect statistically significant (two-sided α=0.025) differences between the placebo group and the 6 dose albendazole treatment group. The same assumptions were made for the 2 dose albendazole treatment regimen so that the same power and sample size considerations apply to the comparison between placebo and the 2 dose albendazole treatment. An alpha value of 0.025 was chosen since two comparisons (placebo vs. 2 doses of albendazole, placebo vs. 6 doses of albendazole) will be performed. Based on prior experience, a drop out rate of approximately 20% is expected, resulting in a sample size of 20 subjects for each of the three treatment arms.

2 STUDY FLOW CHART

		Group 1 (20 subjects)		Group 2 (20 subjects)		Group 3 (20 subjects)	
**Month**	**Day**	**Treatment**	**Examination**	**Treatment**	**Examination**	**Treatment**	**Examination**
**M-2 to 0**	**NA**	**Village information, Informed Consent for initial Screening, Initial Screening**					
			- CBS		- CBS		- CBS
**M-2 0**	**NA**	**Informed Consent for the remainder of the study, if applicable Final subject qualification**					
			- Q-CE<br>- PT<br>- LV		- Q-CE<br>- PT<br>- LV		- Q-CE<br>- PT<br>- LV
**M0**	**NA**	**Final subject selection, randomization Baseline data acquisition**					
			- CBS		- CBS		- CBS
M0	D 0	Placebo	AEs	Albendazole	AEs	Albendazole	AEs
	D 3	-	AEs	-	AEs	-	AEs
	D 7	-	AEs	-	AEs	-	AEs
M2	D 60*	Placebo	- CE<br>- CBS<br>- PT<br>- AEs	Albendazole	- CE<br>- CBS<br>- PT<br>- AEs	Albendazole	- CE<br>- CBS<br>- PT<br>- AEs
	D 63	-	AEs	-	AEs	-	AEs
	D 67	-	AEs	-	AEs	-	AEs
M4	D 120*	Placebo	- CE<br>- CBS<br>- PT<br>- AEs	Placebo	- CE<br>- CBS<br>- PT<br>- AEs	Albendazole	- CE<br>- CBS<br>- PT<br>- AEs
	D 123	-	AEs	-	AEs	-	AEs
	D 127	-	AEs	-	AEs	-	AEs
M6	D 180*	Placebo	- CE<br>- CBS<br>- PT<br>- AEs	Placebo	- CE<br>- CBS<br>- PT<br>- AEs	Albendazole	- CE<br>- CBS<br>- PT<br>- AEs
	D 183	-	AEs	-	AEs	-	AEs
	D 187	-	AEs	-	AEs	-	AEs

		**Group 1 (20 subjects)**		**Group 2 (20 subjects)**		**Group 3 (20 subjects)**	
**Month**	**Day**	**Treatment**	**Examination**	**Treatment**	**Examination**	**Treatment**	**Examination**
M8	D 240*	Placebo	- CE<br>- CBS<br>- PT<br>- AEs	Placebo	- CE<br>- CBS<br>- PT<br>- AEs	Albendazole	- CE<br>- CBS<br>- PT<br>- AEs
	D 243	-	AEs	-	AEs	-	AEs
	D 247	-	AEs	-	AEs	-	AEs
M10	D 300*	Placebo	- CE<br>- CBS<br>- PT<br>- AEs	Placebo	- CE<br>- CBS<br>- PT<br>- AEs	Albendazole	- CE<br>- CBS<br>- PT<br>- AEs
	D303	-	AEs	-	AEs	-	AEs
	D 307	-	AEs	-	AEs	-	AEs
M14	D 420**	-	- CBS<br>- AEs	-	- CBS<br>- AEs	-	- CBS<br>- AEs
M18	D540**	-	- CBS<br>- AEs	-	- CBS<br>- AEs	-	- CBS<br>- AEs

AEs: Adverse events monitoring, laboratory evaluations as clinically indicated
CBS: Calibrated Blood Smear
CE: Clinical examination during follow up, laboratory evaluations as clinically indicated
LV: Laboratory values (alanine aminotransferase, aspartate aminotransferase, creatinine, haemoglobin, complete blood count)
PT: Pregnancy test
Q-CE Clinical evaluation for exclusion criteria, laboratory evaluations
* Day 60, 120, 180, 240, 300: each ±3 days
** Day 420, 540: each ± 1 week

3 RATIONALE AND BACKGROUND

Loiasis is a parasitic infection endemic in the rain forest areas in Sub-saharan Africa caused by the filarial nematode *Loa loa*. The adult worms (females up to 70 mm long with a diameter of around 0.5 mm, males smaller) migrate through the subcutaneous tissues and into the conjunctiva, which has given the disease in many areas the local name 'eye worm'. The microfilaria (up to 300 µm x 8 µm) are present in the peripheral blood during the day time (see Figure 1).

Clinical manifestations include 'Calabar swelling', a hypersensitivity response to the antigenic material released by the macrofilaria, which lasts a few hours or days and is usually painless, itching, swelling of lids with itching and pain, and hydrocele caused by adult worms in the scrotum. In most cases loiasis is a relatively benign disease with a good prognosis and many patients do not have defined signs or symptoms for years before the infection is diagnosed (primarily via the Calabar swelling or the 'eye worm'). It is not know to what extent infection with *Loa loa* causes fatigue, recurrent fever and perhaps arthritic pains. Loiasis appears to be rarely associated with disability or serious ill health. However, since loiasis can be associated with significant and sustained eosinophilia, it may be one of the filarial infections that contributes to

endomyocardial fibrosis due to the effects of eosinophils on the heart (Parry et al. 2004, Duke 1991, Ukety, personal communication). *Loa loa* infected patients frequently complain about itching, swelling of the face, the lip, or the legs, the eye during the passage of adult *Loa loa,* asking for a curative treatment.

However, there is currently no safe treatment to reduce or eliminate *Loa loa* infection.

Diethylcarbamazine (DEC) is effective against *Loa loa* macrofilaria, but can cause serious complications such as meningoencephalitis which is sometimes fatal (Duke 1991).

Ivermectin is an effective *Loa loa* microfilaricide, which results in a very rapid around 80-90% reduction of microfilaraemia within around 3 days of treatment (Richard-Lenoble et al. 1988, Carme et al. 1991, Chippaux et al. 1992, Martin-Prevel et al. 1993). The initial studies conducted on the effect of ivermectin in small numbers of subjects with relatively low levels of *Loa loa* microfilaremia showed that ivermectin was well tolerated in these subjects. However, when large numbers of *Loa loa* infected subjects were treated with ivermectin (Mectizan) within the framework of onchocerciasis control programmes, it was found that ivermectin treatment of subjects heavily infected with *Loa loa* can result in serious adverse events (SAEs), including encephalopathies (see e.g. Figure 1).

Table 1 summarizes data on serious neurological adverse events reported in clinical studies and/or during the implementation of ivermectin mass treatment and provides the pretreatment *Loa loa* microfilaremia (LLM).

Table 1 Overview of severe or serious neurological events reported in the literature

**Adverse event(s) (Comments)**	**Pre-Tx LLM (mf/ml)**	**Pre Tx LLM count based on**	**Source**
Grade 2 coma<br>(1/26 subjects with >30000mf/ml)	163000	Pretreatment measurement	Ducorps et al. 1995
Serious neurological reaction (disorders of consciousness, coma, incontinency, sensory deficits, hypertonia)<br>(2/>3242 subjects with *LLM* including 18 with geometric mean *LLM* of 83448 mf/ml, 2/160 subjects with LLM >30000) [1]	50502<br>152940	Pretreatment measurement	Gardon et al. 1997
PLERM [2]	36000	Extrapolated from mean post-treatment level	MEC 2002

[1] 17877 subjects were treated. *Loa loa* blood microfilaremia was evaluated in 5500 (31%) subjects, including 18/20 with serious neurological reaction, of whom 58% (3242) were *Loa loa* positive (Gardon et al. 1997) and 160 (2.9%) had LLM >30000 mf/ml pre-treatment (Boussinesq et al. 1998).

[2] PLERM: Probable or possible *Loa loa* encephalopathy related to treatment with Mectizan. *Loa loa* encephalopathy related to treatment with Mectizan: Encephalopathy (without seizures, usually with fever) in a person previously healthy and without another underlying cause for emcephalopathy, onset of progressive CNS symptoms and signs within 7 days of treatment with ivermectin, illness progressing to coma without remission with assessment of probability of relationship to Mectizan treatment based on availability of LLM data: Probable if peripheral blood *L.loa* >10000 mf/ml pretreatment or >1000 mf/ml within 1 month post treatment or >2700 mf/ml within 6 months of treatment, and/or *L.loa* microfilarae in the CSF. Possible if semi-quantiative or non-quantitative positive (i.e. +, +, +++) *L.loa* microfilariae in pheripheral blood within 1 month post treatment.

The relatively low number of subjects with high LLM for which reliable LLM and adverse event data are available from prospective studies (Ducorps et al. 1995, Gardon et al. 1997) and the likely underreporting of SAEs and lack of pre-treatment LLM values from mass treatment with ivermectin (MEC 2002, Twum-Danso, 2005), do not allow to definitively determine the minimum

pre-ivermectin treatment LLM that puts subjects at risk for development of serious and/or severe adverse events.

Minimum levels proposed based on the available data include:

- > 50000 mf/ml results in an odds ratio of >1000 for serious adverse reactions (including non-neurological reactions, Gardon et al. 1997)
- > 50000 mf/ml for encephalopathy (MEC 2002)
- > 50000 mf/ml for disorders of consciousness (Boussinesq et al. 2003)
- > 36000 mf/ml (approximately) for PLERM (MEC 2002)
- > 30000 mf/ml, probability of >0.007 for development of serious adverse reactions, whether neurological or not (Boussinesq et al. 2003)

A pre-treatment LLM of > 10000 mf/ml as the criterion for the diagnosis of probable LERM was established in 1995 by a consultation and not revised to date (MDP, 1996).

Analysis of the most comprehensive prospectively obtained data available to date on the adverse reactions to ivermectin in subjects with LLM quantitated prior to ivermectin treatment resulted in the conclusion that the relative risk of developing any type of adverse reaction resulting in functional impairment requiring assistance for everyday natural functions and household activities for at least several days ('marked reactions') is statistically significantly higher in subjects with >8100 mf/ml than in subjects without detectable LLM (Gardon et al. 1997).

Disease control programmes for onchocerciasis rely on the community directed mass treatment of ivermectin (CDTI). During CDTI, subjects living in areas hyper- or mesoendemic for onchocerciasis are being administered a single dose of ivermectin without the presence of health care personnel and independent of whether or not they are infected. This is possible because of the excellent safety profile of ivermectin in healthy subjects and subjects infected with *Onchocerca volvulus* and not co-infected with *Loa loa*.

Mass treatment procedures are also in place for lymphatic filariasis (LF) control. The objective of the global program for the elimination of lymphatic filariasis (GPELF) is to interrupt transmission of the disease by treating the entire at risk population with a single administration of two drugs given simultaneously once yearly. Where onchocerciasis and LF are co-endemic in Africa, mass treatment uses ivermectin combined with albendazole. In areas in Africa and in Asia where lymphatic filariasis is not co-endemic with onchocerciasis, albendazole is combined with DEC. The Safety Review Committee which reviewed the adverse event data obtained during active surveillance in 12 countries instituting mass drug administration (MDA) for LF control decided that there was no need for further active surveillance for adverse events during MDA (WHO, 2003).

The risk of serious and /or severe adverse events among *Loa loa* infected subjects treated with ivermectin restricts onchocerciasis disease control efforts in two ways:

- As per the recommendations of the Mectizan^{R} Expert Committee (MEC) and the Technical Consultative Committee of the African Programme for Onchocerciasis Control (APOC), mass treatment in areas with high prevalence or intensity of infection with *L. loa* requires considerable additional operational investment (education of target population, training and preparation of medical personnel on SAE management, equipment of health care facilities for SAE treatment, employment of medical personnel for population surveillance). This requires substantial additional resources (Mectizan Expert Committee and The Technical Consultative Committee, June 2004) which are not available for further expansion of onchocerciasis control.

- The fear of serious and/or severe adverse events leads to a reduced participation of the population in CDTI. As high a population coverage as possible is, however, not only in the interest of individual subjects with onchocerciasis, but also in the interest of the community and region since it is the key to reduction of disease transmission.

The impact on lymphatic filariasis (LF) control efforts is even greater:

- Following the experiences and analysis of SAEs during onchocerciasis control, and a risk-benefit assessment, it was decided not to move LF control into areas co-endemic with loiasis. Thus, the progress of LF control is completely stalled in areas co-endemic for LF and loiasis.

A treatment reducing the *L. loa* microfilaremia well below the currently identified risk level and safe enough to be administered in a community directed way prior to CDTI or MDA for LF could allow standard CDTI to proceed and LF control to be instituted in *Loa loa* co-endemic areas. However, no safe treatment that reduces L. loa microfilaremia is available.

The mechanism by which ivermectin treatment in subjects with high LLM leads to serious neurological adverse events is unknown. Based on the analysis of the symptoms observed, prior experience with DEC treatment associated encephalopathies in subjects with high LLM and the knowledge of Mazzotti reactions following ivermectin or DEC treatment of subjects with onchocerciasis, three potentially concomitant mechanisms have been proposed: (1) obstruction of the cerebral microcirculation in consequence of massive amounts of paralyzed or dead *Loa loa* microfilariae, (2) penetration of live microfilaria into the brain tissue, (3) inflammatory processes in the brain (Boussinesq 2003).

Independent of the mechanism and the presence and relative importance of potential co-factors, serious neurological reactions following ivermectin treatment have to date been observed only in subjects with high LLM (Table 1, Figure 1). Consequently, a safe treatment for reducing LLM should have no or very inefficient *Loa loa* microfilaricidal activity and reduce LLM exclusively or primarily through a macrofilaricidal or macrofilaria sterilizing effect so that the reduction in LLM occurs slowly primarily through *Loa loa* microfilaria reaching the end of their natural life span (unknown, but estimated at up to 6 months based on data from human *Loa loa* infected monkeys, Duke 1960) and not being replaced. Based on prior data on the effect of albendazole on LLM (see further below and Table 2), albendazole may have the efficacy and safety profile required.

Albendazole is a benzimidazole. Benzimidazoles are broad-spectrum anthelmintics with a high therapeutic index whose primary site of action are the microtubules via inhibition of tubulin polymerization.

Due to its low aqueous solubility, oral bioavailability of albendazole is poor (<5%). Following absorption, albendazole is quickly metabolized into the active metabolite albendazole sulphoxide which is further metabolized to albendazole sulfone and other primary oxidative metabolites.

Urinary excretion of albendazole sulfoxide is a minor elimination pathway, accounting for less than 1% of the dose recovered in urine. Consequently, while pharmacokinetic studies in patients with impaired renal function have not been conducted, it is unlikely that clearance of albendazole and its primary metabolite, albendazole sulfoxide, would be altered in these patients (Glaxo Smith Kline 2001).

After administration with a fatty meal several fold higher C_{max} and four fold higher $AUC_{0\text{-}36h}$ values were obtained than in subjects who took the same dose in the fasting state. A single 800

mg dose of albendazole administered with a fatty meal resulted in an albendazole sulphoxide C_{max} of 1943 ± 403 µg/L, and $AUC_{0\text{-}36h}$ of 20992 µgh/L (Awadzi et al. 1994).

Pharmacokinetic data from subjects infected with *O. volvulus* administered 200 mg albendazole q6h for 3 days or 200 mg albendazole q4h for 3 days suggest that the major fraction of the albendazole sulphoxide detectable during the first three days after the first dose is due to the doses administered during the first day (Awadzi et al. 1994). This is consistent with data that show that following 4 weeks of treatment with 200 mg albendazole TID, albendazole sulfoxide concentration was 20% lower than during the first half of the treatment period (Glaxo Smith Kline, 2001).

The safety data from use of albendazole for anthelminthic treatment from clinical trials as well as the published literature and/or spontaneously reported to the manufacturer were recently reviewed (Horton 2000).

After dosing for ≤3 days (400 mg single dose, 400 mg QD for 3 days or 800 mg single dose), the incidence of adverse events among 22810 subjects in different clinical trials for treatment of intestinal helminths did not exceed 0.386% for any individual type of adverse event. Different types of gastrointestinal effects were the most frequent AEs with a total incidence across all types of <1%. Laboratory value based adverse events included leucopenia (0.044%), raised liver enzymes (0.035%), low red cell count and proteinuria (0.009% each), anemia and raised blood urea (0.004% each). These symptoms are common in the communities with helminth infections so that drug relationship of the AEs is hard to assess. The only study that compared albendazole and placebo treatment in sufficient number of patients did not find significant differences between the two treatment arms (Horton 2000).

In contrast to the very short treatments used for antihelminth or LF indication, and planned in this study (single 800 mg dose, every 2 months up to 6 times), long term daily use of up to 800 mg/day as for hydatic disease (recommended dose regimen: three 28 day cycles of 15 mg/kg/day up to 400 mg BID, with 14 days between cycles) is associated with a higher frequency of AEs. Liver and biliary AEs were the most frequent AE observed in 10.8% among 3282 subjects treated for hydatid disease (Horton 2000). Mild to moderate increases in transaminase levels at least possibly or probably related to albendazole were reported for 15.6% of hydatid disease patients in the GSK safety data base. Upon discontinuation of albendazole treatment, the transaminase values returned to normal. For subjects treated for hydatid disease or for neurocysticercosis (treatment regimen 15 mg/kg/day up to 400 mg BID for 8-30 days) liver function tests before start of each treatment cycle and at least every 2 weeks during treatment and discontinuation of treatment if enzyme levels increase significantly is recommended. Patients with abnormal liver function tests prior to initiation of albendazole treatment should be carefully evaluated since the drug is metabolized by the liver and has been associated with hepatotoxicity in a few patients. (GSK 2001).

Among 11 men with loiasis who received 200 mg albendazole BID for 21 days none had elevations of alanine aminotransferase or aspartate aminotransferase or changes in haematocrit. Adverse events included myalgia (40%), arthralgia (50%), pruritus (20%) (observed in the placebo group in 36%, 36% and 36%, respectively), calabar swelling (20%) and dizziness (10%) (Klion et al 1993).

For the hydatic disease patients , blood counts monitoring is recommended by the manufacturer at the start of each 28 day cycles and every 2 weeks during each 28-day cycle because reversible reductions in total WBC have been observed in <1% of patients and granulocytopenia, agranulocytosis or pancytopenia in rare cases (GSK 2001).

There is one case report from physicians in Switzerland on an encephalopathy in a subject with loiasis from Cameroon treated with albendazole in Switzerland (Blum et al. 2001). The PI and TDR consulted with clinical experts (involved in the analyses of encephalopathy cases observed in *L.loa - O. volvulus* co-infected patients and in treatment of subjects with numerous antihelminths, including albendazole), on the probability of this encephalopathy being causally related to the albendazole treatment this subject received and consequently on safety monitoring in this study. There was unanimous agreement that the probability that this encephalopathy was causally related to *L.loa* infection was extremely remote since (1) the *L. loa* microfilariaemia was extremely low (152 mf/ml), (2) the medical history of the subject provided likely causes (diabetic with antecedent unexplained neurologic signs) and (3) the time course of the symptoms is completely inconsistent with the slow effect of albendazole on microfilarial levels. The experts advised that the safety monitoring planned in this protocol is adequate.

Previous trials with albendazole (Table 2) have shown that administration of albendazole over a short period of time (one to three days) does not result in a significant, sustained reduction of LLM. Administration over 21 days was more effective, but is not feasible within mass treatments. Furthermore, the study evaluating 21 days of albendazole treatment did not include subjects with more than 30000 mf/ml.

Table 2 Overview of effect of albendazole treatment on *Loa loa* microfilaremia

Albendazole regimen (number of subjects)	Pre-Tx LLM (mf/ml)<br>Time of measurement<br>Range (Geom. Mean)	Efficacy (% geometric mean preTx values)	Adverse events
200 mg BID, 21 days (11)[1]	11:30-14:30<br>385 - 20200 (2369)	- 70%-20% over 3 mos post Tx start<br>- 20% at 6 mos	Myalgia, arthralgia, pruritus, increased appetite, dizziness, calabar swelling
Placebo BID, 21 days (12)	236 - 27500 (3119)	- 37%-192% over 3 mos post Tx start<br>- 84% preTx at 6 mos	Myalgia, arthralgia, pruritus, increased appetite
600 mg, single dose (23)[2]	11:00 - 13:00<br>7 - 1377 (180)	- 60%-125% over 3 mos post Tx start<br>- 60% at 6 mos<br>- 57% at 10 mos	No adverse events reported
Mebendazole, 100 mg BID, 3 days (24)	3 - 5535 (195)	- 70%-132% over 3 mos post Tx start<br>- 93% at 6 mos<br>- 86% at 10 mos	
400 mg, QD, 3 days (48)[3]<br>- 180 days FU (37)<br>- placebo (28)<br>- 180 days FU (27)	10:00 - 15:00<br>100 - 33837 (2550.3)	ALB: 90%-70% over 3 mos post Tx<br>90% at 6 mos<br>Plac: 105%-95% over 3 mos post Tx<br>>120% at 6 month	No difference between albendazole and placebo treatment
Placebo, QD, 3 days (51)<br>- 180 days FU (40)<br>- 400 mg, QD, 3 days (36)<br>- 180 days FU (28)	164.3 - 24050 (5507.9)	Plac: 98%-110 over 3 month post Tx<br>120% at 6 mos<br>ALB: 95%-65% over 3 mos post Tx<br>90% at 6 mos	
400 mg, BID, 3 days (47)[4]	11:00-15:00<br>260 - 54180 (3800)<br>Subgroup of 12 with LLM >8000<br>8460 - 54180 (18594)	All subjects<br>71%-82% over 3 mos post Tx<br>91%-81% 6-9 mos post Tx<br>> 8000 PreTx<br>94-53% pre Tx over 3 mos post Tx<br>104%-89% 6-9 mos post Tx	Itching, abdominal pain, diarrhea Increased ALT/AST on day 5
Multivitamin	240-46780 (3467)<br>Subgroup of 14 with LLM>8000<br>8640-46780 (15558)	All subjects:<br>98-83% over 3 mos post Tx<br>91-107% 6-9 mos post Tx<br>>8000 preTx<br>92-74% over 3 mos post Tx<br>82-114% 6-9 mos post Tx	Itching, diarrhea

1 Klion et al. 1993, 2 Kamgno, Boussineq 2002, 3 Tabi et al. 2004 (% in efficacy cell approximated from Figure 2 in the publication). 4 Tsague-Dongmo et al. 2002

The time course in LLM seen in these studies, comparison of the effect of different doses evaluated as well as significant intra-individual variation in the effect of a single albendazole

dose, suggest that multiple exposure of the *Loa loa* macrofilaria with albendazole at two month intervals may have the desired effect on the reproductive capacity of the macrofilaria. The safety data available show that a single dose of 800 mg has an excellent safety profile, consistent with other data on the safety of single doses of albendazole (see above).

This study will evaluate the effect of an albendazole dose of 800 mg, administered every two months 2 or 6 times, respectively.

4 STUDY GOAL, OBJECTIVES AND EXPECTED OUTCOMES

The goal of this study is to evaluate whether one or both of the evaluated albendazole treatment regimens are sufficiently effective in reducing *L.loa* parasitemia and safe to potentially be qualified as candidate for mass treatment for the reduction of LLM.

The primary objective of this study is to evaluate the efficacy of two different albendazole treatment regimens in sustainably reducing *L. loa* microfilarial load in subjects with high pre-treatment microfilaremia.

The secondary objective is to assess the safety of these treatment regimens in the study subjects in terms of frequency of adverse events by type and severity.

In case this proof of principal study shows that one or both of the two albendazole treatment regimens, but not the placebo treatment reduce microfilaria counts sustainably (i.e. for a period of at least 4 months) by ≥ 50% of pretreatment values, and the safety profile is, as expected based on the prior experience with albendazole, consistent with the requirements for mass treatment, the results will be discussed with the stake holders to decide on further studies to be conducted.

5 STUDY DESIGN

5.1 Overview

This is a randomized, double blind, placebo controlled field-based study of subjects infected heavily with *L. loa* who are otherwise of good health and live in an area not endemic for onchocerciasis or lymphatic filariasis. An area not co-endemic with onchocerciasis was selected because all areas endemic for onchocerciasis in Cameroon are either already conducting or are initiating CDTI and ivermectin treatment reduces *Loa loa* microfilaremia (see section 3) making it impossible to find subjects with high LLM. Furthermore, since ivermectin is an effective *Loa loa* microfilaricide, the effect of albendazole on LLM can only be evaluated if during the study period the subjects do not take ivermectin. Enrolling subjects co-infected with *Onchocerca volvulus* in this study would mean that for the around 20 months duration of the study, these subjects would be deprived of the benefit of ivermectin treatment, which should be administered at least on a yearly to maintain *Onchocerca volvulus* microfilaria levels below those where symptoms and potentially disease progression occur.

Selection of an area endemic for lymphatic filariasis and highly endemic for loiasis is not possible, because the data from the recently conducted survey for the distribution of lymphatic filariasis in Cameroon have been deemed to be unreliable.

While the medical need for a LLM reducing treatment is greatest among populations living in areas co-endemic for loiasis and onchocerciasis and/or lymphatic filariasis (see section 3), populations living in areas endemic only for loiasis will also benefit from a treatment that safely reduces LLM and thus presumably reduces the signs and symptoms and potentially long-term sequelae of loiasis. Among these populations particularly the subjects with high *Loa loa*

microfilaremia, such as those selected for this study, will benefit from the qualification of an LLM reducing treatment.

60 males and non-pregnant females between 18 and 65 years with an LLM of >15 000 mf/ml will be enrolled in this study and randomized by pretreatment LLM to one of three treatment regimens:

- Placebo Control (Group 1)
  these subjects will receive 6 oral doses of placebo, one every two months
- 2 doses of albendazole (Group 2)
  these subjects will receive 2 oral doses of albendazole (800 mg) 2 months apart, followed by four doses of placebo every two months
- 6 doses of albendazole (Group 3)
  these subjects will receive 6 oral doses of albendazole (800 mg), one every two months

Prior to each administration, subjects will undergo a pregnancy test, if applicable, as well as a clinical examination, quantitation of *L.loa* parasitemia and be asked about any adverse events since the last visit. If they show signs and symptoms of intestinal helminths, the stool will be examined. If clinically indicated or indicated based on the report of adverse event(s) by the subject, a full blood count and laboratory evaluations will be performed as appropriate. Follow up for adverse events will be conducted at the day of treatment, day 3 and day 7 after each treatment as well as at the next treatment and/or efficacy follow up visit.

Efficacy will be evaluated via microfilaria counts in a calibrated blood smear obtained prior to each administration as well as 4 months and 8 months after the last dose was administered. To account as much as possible in a field based study for the effect of the diurnal periodicity of the *L. loa* counts in the peripheral blood (), the calibrated blood smear samples will be obtained between 11:00 and 15:00 and at around the same time of day in each village.

Figure 1 ***Loa loa* microfilaremia counts and diurnal change in *Loa loa* infected subjects treated with ivermectin in Cameroon**

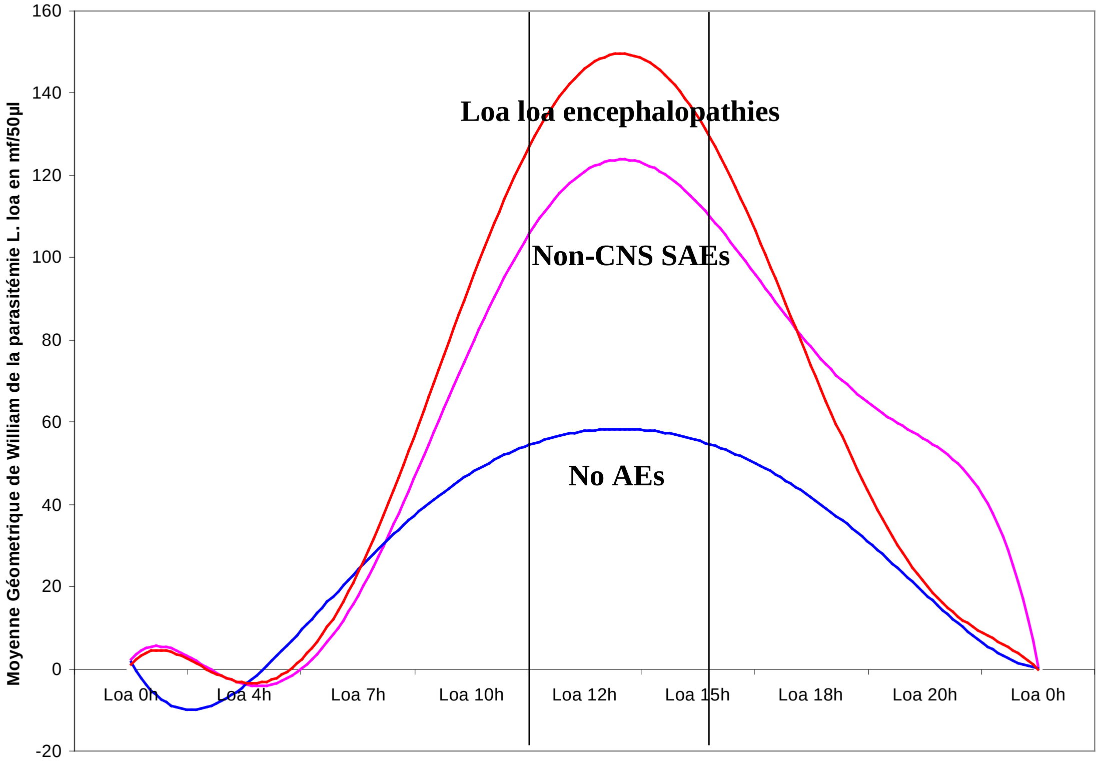

Non-CNS SAE: serious adverse events not affecting the central nervous system.

Following unblinding of the data and completion of the analysis, the villages will be informed about the outcome of the trial and what consequences can be drawn for further studies to qualify albendazole for treatment of loiasis.

5.2 Primary endpoint

The primary endpoint will be the proportion of subjects in whom there is a sustainable (≥ 4 months) reduction in microfilaremia by ≥50% of pretreatment values at any time point post the first drug administration by treatment group.

5.3 Secondary endpoints

- Proportion of subjects whose microfilaria counts have been sustainably reduced to <8100 mf/ml by treatment group
- % of reduction in microfilaremia (range, median, Williams geometric mean) at each time point by treatment group by microfilaria level pretreatment and gender
- Microfilaria loads at each time point in each treatment group, compared by non-parametric tests because of the non-normal distribution of the *Loa* microfilaremia.
- Evolution of microfilaria counts in time

- Type, frequency and severity of adverse events (all adverse events and separate only those with a reasonable probability of relationship with study drug, see section 8.5).

6 METHODOLOGY

6.1 Selection of study population and acquisition of baseline data

The trial will be carried out in the South and Central provinces of Cameroon. Subjects will be recruited from villages in the Nyong and Soo and Mvila division. These areas are endemic for loiasis but without onchocerciasis and without lymphatic filariasis.

The schematics below provides an overview of the selection of the study subjects.

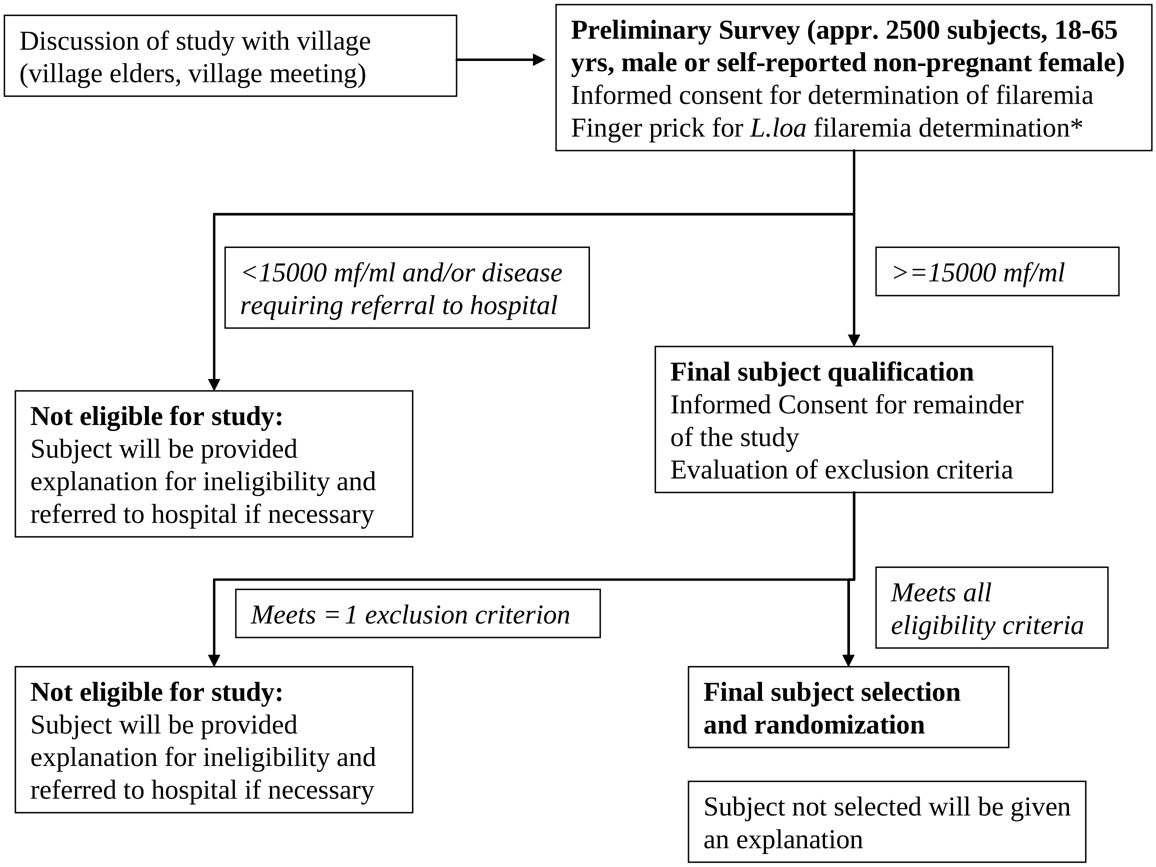

* A general medical consultation will be offered to those who have medical complaints (including villagers who want medical consultation but do not want to participate in the preliminary survey for LLM) and some drugs will be available to treat subjects with any ailment that is amenable to treatment. Villagers will be referred to a health center if the ailment is not amenable to treatment by the study team. These examinations will not yield any data for the study.

6.1.1 Inclusion criteria

Subjects who qualify for this study have to fulfill ALL of the following criteria:

- 18 to 65 year old male or female.

- *L. loa* microfilaremia >15 000 mf/ml as determined by calibrated blood smear.
- Do not plan on moving out of the area over the next two years.
- Given informed consent (written, witnessed, signed or thumb printed).

6.1.2 Exclusion criteria

Subjects who meet ANY of the following criteria will be excluded from the study

- Treatment with a benzimidazole during the last 12 months
- Self-reported allergy to benzimidazoles.
- Pregnancy, assessed by urine pregnancy test (β-HCG) before each treatment in all women of child-bearing potential.
- Clinical signs and symptoms and laboratory evidence of intestinal helminths
- Any serious underlying medical condition
- Past or current history of neurological or neuropsychiatric disorders
- Clinical and/or laboratory evidence of significant liver disease, kidney disease or anaemia or any other condition that in the investigator's judgment should exclude the subject from the study.

6.1.3 Conduct of screening

Based on the prevalence of *L. loa* in these areas and the inclusion / exclusion criteria, it is estimated that between 2% and 5% of the population is eligible for this study. Thus in order to recruit 60 subjects for the study up to around 2500 subjects need to be screened. It will be necessary to conduct screening in around 25 communities, considering the average of around 100 18-65 year old persons per community.

Given the large number of subjects to be screened, screening will be conducted in two steps:

1. Preliminary survey among 18-65 year male villagers and 18-65 year old female villagers who present themselves as not pregnant to identify subjects who fulfill the LLM inclusion criterion
2. Definitive subject identification by assessment of the presence of any of the exclusion criteria among subjects with > 15000 mf/ml based on the preliminary survey.

6.1.3.1 Village information about study and village focal point

Prior to the initiation of the preliminary survey, each village in the study area will be visited to inform the villagers about the objectives and procedures of the study, to inform them about and discuss with them the risks and benefits of study participation and to answer any questions they may have. If villagers are interested in participating in the study, the time of day for the visits during the study will be agreed upon. A person in the village will be identified during the discussions with village elders and villagers who will serve as the local focal point for the study within the village.

6.1.3.2 Preliminary survey

During the preliminary survey, males and non-pregnant (based on 'self-assessment') females in each community meeting the age inclusion criterion will be invited for a *Loa* microfilarial load assessment.

Prior to the initiation of each volunteer's screening for Loa loa, the informed consent information for this part of the study will be given to the potential volunteers to read or will be read to them (as per their preference), questions answered and informed consent obtained via signature or

thumb print. The common language used in this part of Cameroon is French. Both genders are legally adults in Cameroon at 18.

Each subject who gives informed consent will be given a subject number. In case report forms, the subjects name will not be used, only subject number and initials.

During the preliminary survey, subjects will:

- Have a blood sample (50 µl via finger prick and capillary tube) taken for parasitological examination. The blood sample will be labeled with the date and time of collection and subject number.

Given the diurnal variation in blood microfilaria levels (Kamgno & Boussinesq, 2002, Figure 1), blood samples for screening (as well as baseline and for efficacy follow up evaluations) will be obtained between 11:00 and 15:00 o'clock. All possible efforts will be made so that for each village the examination time will be about the same throughout the study.

The date and time of day of the collection of a blood sample for each subject will be recorded.

During the preliminary survey, a general medical consultation will be offered to those who have medical complaints (including villagers who want medical consultation but do not want to participate in the preliminary survey for LLM) and some drugs will be available to treat subjects with any ailment that is amenable to treatment in the village.

Subjects who are not eligible for the subsequent part of the study will be given the explanation this.

6.1.3.3 Definitive subject qualification for the study

All subjects who have >15 000 *L. loa* microfilaria/ml based on the preliminary survey will be included in the definitive subject identification activities via evaluation for exclusion criteria if they consent to it. Prior to this section of the study, the informed consent information for the remainder of the study will be given to these subjects to read or will be read to them (as per their preference) and the study will be discussed with them, questions answered and informed consent obtained via signature or thumb print.

For the definitive subject qualification, subjects will undergo

- A clinical examination to assess presence of any serious underlying medical condition, clinical signs and symptoms of intestinal helminths, clinically evident liver disease, kidney disease or anaemia. In subjects with clinical signs and symptoms of intestinal helminths (abdominal pain, diarrhoe, vomiting, anaemia) a stool examination for helminths will be conducted)
- Questioning about treatment with benzimidazole during the past 12 months
- Questioning about allergy to benzimidazoles
- Questioning for past or current history of neurological or neuropsychiatric disorders
- Pregnancy test on a urine sample (β-HCG) (all women of childbearing potential)
- Venous blood sampling (4 ml) for transaminase activity (Alanine aminotransferase (ALT, SGPT), aspartate aminotransferase (AST, SGOT), creatinine levels, haemoglobin levels and complete blood count (CBC)**.**

6.1.3.4 Definitive subject selection

Whether or not a subject's microfilaria count drops by ≥ 50% may depend on the initial microfilaria burden. Of particular interest for this study are those, relatively rare, subjects with pretreatment LLM of >30000 mf/ml since those are the ones most likely to experience signs and

symptoms of loiasis and potential long term sequelae of loiasis and are also those at highest risk for SAEs upon ivermectin treatment (see Table 1).

Thus, if more than 60 subjects have qualified for participation in the study based on evaluation of inclusion and exclusion criteria, the 60 subjects with the highest microfilaria counts will be selected for the study (see section 6.2.4.2 and section 10).

Subjects who are not eligible or are not being selected for inclusion in the study will be given an explanation for the reason of their exclusion and referred to medical attention if indicated.

6.1.3.5 Baseline LLM in subjects selected for study participation

On the day of the first treatment, subjects selected for study participation will have a blood sample (50 µl via finger prick and capillary tube) taken for parasitological examination for baseline microfilaria counts (*Loa loa* and other parasites in the blood, if applicable, e.g. *Mansonella perstans)*. For subjects in which the preliminary survey indicated LLM above 30000 mf/ml, the blood sample will be distributed over two slides for accurate quantitation of baseline LLM. The blood samples will be labeled with the date and time of collection and subject number.

6.1.3.6 Subject information on screening results

Subjects will be informed individually verbally of the results of the examinations performed on them and also provided cards with their name (and photo unless they do not want one) and the results of all tests performed as well as all treatments administered based on the results of the examinations performed during the screening.

For subjects who participate in the study, these cards will be updated with any results relevant to their medical history that may be obtained during the examinations performed during the course of the study.

6.1.4 Subject identification and screening log

A record of all subjects screened will be maintained (screening log). The screening log will contain the following information:

- Date of screening
- Subject name
- Subject initials
- Subject number
- Village
- Date of birth (or age if date of birth unknown)
- Gender
- Passed or failed screen
- Reason for screen failure (if applicable)

6.1.5 Screen failures

Subjects who sign or thumbprint an informed consent form but are found during screening not to qualify for the study are regarded as screen failures and the reason for the screen failure will be recorded in the screening log.

Subjects who do not qualify for the study or were not selected despite fulfilling all inclusion and no exclusion criterion, will be provided individually, verbally an explanation for the reason of their exclusion from the study.

If subjects who fail screening were discovered during the preliminary survey to have *L.loa* (or *Mansonella perstans* another parasite in the blood), they will be informed about this. There is no safe and effective treatment for either *L. loa* infection or *Mansonella perstans*.

If subjects were discovered during the screening examination to have a disease that needs treatment beyond the capacity of the study team, they will be informed about their disease and referred to medical attention for consultation and treatment. A district medical officer is part of the study team.

6.1.6 Subject withdrawal

6.1.6.1 Withdrawal from the study

Subjects will be told during the informed consent process that they can withdraw from study treatment or from the whole study at any time.

Subjects who want to withdraw from the whole study at any time after the first treatment will be asked to undergo one last clinical examination and examination for adverse events.

The investigator will withdraw any subject from the study at any time if he feels that that is in the best interest of the subject.

6.1.6.2 Withdrawal from study treatment

Subjects will be withdrawn from further treatments if they become pregnant or develop another exclusion criterion with the exception of intestinal helminths, experience a serious adverse event, or develop any other medical conditions that in the investigator's opinion should result in study drug withdrawal.

Subjects who are diagnosed with intestinal helminths during the study will be withdrawn only from the next study treatment and receive 800 mg of albendazole. For subsequent scheduled treatments, these subjects will again receive the blinded treatment they were randomized to.

Subjects who are withdrawn from study treatment but not the study will continue to be followed up until the last scheduled efficacy follow up.

6.1.6.3 Reasons for withdrawal

For a mass treatment with albendazole to reliably reduce LLM to levels that do not expose *Loa loa* infected subjects to risk of severe and/or adverse events upon mass ivermectin treatment for onchocerciasis or LF control, compliance of all individuals in a community is key. Compliance with a multiple dose treatment with doses administered in several month intervals is a major challenge for mass treatments. To enable appropriate information, education and motivation for compliance to be implemented in the mass treatment schemes it is important to understand reasons for non-compliance.

To obtain compliance related data that can be used to appropriately design further evaluation of multiple dose albendazole regimen(s) for mass treatment not only in terms of efficacy and safety, but also in terms of field implementation, subjects who withdraw their consent to further study treatment or further participation in the study, and subjects who do no participate in all scheduled treatment days, will be asked to provide the reasons. During the informed consent process it will be explained to subjects not only that they have the right to withdraw from the study at any time, but also that they have no obligation to answer the question for the reason of their withdrawal.

6.1.7 Description of methods used during screening and baseline data acquisition

6.1.7.1 Parasitological examination

The method of parasitological examination at screening and baseline data acquisition as well as at each efficacy follow up visit (2, 4, 6, 8, 10, 14, 18 months post first treatment) will be the calibrated blood smear (CBS). This method is to date the best quantitative examination for LLM. It has been used in all trials related to loiasis, and for the basis/validation for RAPLOA, the method now used by the onchocerciasis control programs to identify areas with high prevalence of loiasis.

This examination will be done by finger prick after a careful cleaning of the finger. The blood will be collected with a 50µl non-heparinized capillary tube, the 50 µl spread on a labeled slide, dried and Giemsa stained and dried at ambient temperature. All *L. loa* and *Mansonella perstans* microfilariae on the slide will be counted at a magnification of 100. *L. loa* and *Mansonella perstans* microfilariae are distinguished primarily based on size, since *M. perstans* microfilaria are approximately half the size of *Loa loa* microfilaria. When necessary correct identification will be verified via the membrane surrounding *Loa loa* microfilaria which becomes visible at higher magnification (400 or 1000).

All slides will be read by the same technician throughout the study with subject number, data and time of sample (but not treatment assignment) known to the technician and again at the end of the study with the technician blinded to subject number, date and time of sample. The result of the blinded reading will be used for data analysis. If the counts obtained for a specific sample during and at the end of the study differ by >10%, the slide will be read again. The mean of the two blinded readings performed after the end of the study will be used for the data analysis.

6.1.7.2 Clinical examination

The clinical examination (CE) to be conducted prior to each treatment (see section 6.2.1) will include medical history pertinent to inclusion/exclusion criteria, the examination of the conjunctivae, temperature, blood pressure, auscultation of the heart and lung, and palpation of the abdomen.

6.1.7.3 Laboratory evaluations

Alanine aminotransferase activity, aspartate aminotransferase activity, creatinine levels and hemoglobin levels will be determined using a Reflotron.CBC will be obtained automatically using am ABX PENTRA 120.

Pregnancy test will be done using urine analyzed with HCG urine/serum test from AMS France.

6.2 SUBJECT TREATMENT

6.2.1 Study drugs and treatment regimen

Albendazole 200 mg tablets and matching placebo will be provided by GSK. 4 tablets of active or placebo will be administered to each subject.

At 0, 2, 4, 6, 8 and 10 months, each subject will be administered after a standard fatty meal (one wheat fritter of 45-50 g filled with approximately 25 g of butter, commonly eaten by the local population and provided to participants as part of this study) orally either 800 mg of Albendazole or placebo, depending on the treatment group they have been randomized to.

Time	Group 1	Group 2	Group 3
Month 0	Placebo	Albendazole 800 mg	Albendazole 800 mg
Month 2	Placebo	Albendazole 800 mg	Albendazole 800 mg
Month 4	Placebo	Placebo	Albendazole 800 mg
Month 6	Placebo	Placebo	Albendazole 800 mg
Month 8	Placebo	Placebo	Albendazole 800 mg
Month 10	Placebo	Placebo	Albendazole 800 mg

6.2.2 Choice of control treatment

Three of the studies conducted to date evaluated the effect of different dosing regimens of albendazole on loiasis microfilaremia in comparison to no treatment.

The data from the subject groups who received no treatment show that *Loa loa* microfilaremia can vary significantly over time without any treatment. Klion et al. (1993) reported that geometric mean microfilaria levels in the placebo group varied between 32% and 192% of pretreatment levels over the 6 months follow up time without a consistent trend and including a drop from 192% to 84% of pretreatment values within a period of 3 months. The data from the study of Tsague-Dongmo et al (2002) showed among subjects with pretreatment levels >8000 mf/ml variations of microfilaria levels of between 73% and 114% of pretreatment values over a period of 9 months, including a drop by 30% over a period of 30 days (see Table 2).

The inclusion of a placebo group is consequently required to assess whether any of the two albendazole treatment regimens is effective, i.e. to see whether any changes in the treatment arms receiving albendazole are actually albendazole-associated and/or larger than the natural longitudinal variation of microfilaria counts as observed in the placebo group. This in turn is the prerequisite for planning further steps to qualify a multiple dose albendazole regimen for reduction of LLM on a community treatment basis.

6.2.3 Determination of compliance

All drugs will be administered to the subjects by study personnel. Thus, compliance will be directly observed.

Date and time study drug was taken will be recorded.

6.2.4 Measures to minimize bias

6.2.4.1 Blinding during treatment and unblinding in case of emergencies

Treatment will be double-blinded (matching albendazole and placebo tablets) and subjects will be randomized to reduce bias in data collection and interpretation.

Given the safety record of albendazole and the fact that the product label for indications that require 400 mg bid dosing of albendazole for up to 30 days, recommends symptomatic treatment (gastric lavage, activated charcoal) and general supportive measures in case of overdose, it unlikely that unblinding the treatment of a subject during the trial will be necessary.

If against all expectations, unblinding will be necessary, the pharmacist who prepared the study medication (see section 6.2.5), and is not otherwise associated with the study, will provide the treatment assignments to the investigator. This person will also provide the randomization codes to the GSK Safety / Case Management Group for unblinded reporting of SAEs to the regulatory authorities:

GSK Safety Group	Contact Person	Helen Goumeas
	Fax number:	+44 (0)208 4239534
	Phone number:	+44 (0)20 8966 2256
	e-mail:	Helen.Q.Goumeas@gsk.com

The sponsor will be informed within 3 days if the treatment of a subject was unblinded, the reason for the unblinding and the actions taken after unblinding.

6.2.4.2 Randomization

Whether or not a subject's microfilaria count drops by ≥ 50% of pretreatment values may depend on the initial microfilaria burden.

Of particular interest for this study are those, albeit rare, subjects with pretreatment LLM of >30000. Thus, if more than 60 subjects have qualified for participation in the study, the 60 subjects with the highest microfilaria counts will be selected for the study and randomized taking into account the requirement for similar distribution of pre-treatment LLM in the three treatment arms. At least 5 of the 20 subjects enrolled in each treatment arm should have a screening LLM of >30000 mf/ml.

To take into account that the drop in LLM may be dependent on the pre-treatment LLM, randomization will be by strata defined by the pre-treatment microfilaremia level obtained at screening, see section 6.1.3.2. The microfilaremia strata will be:

- 15 001 to 30 000 mf/ml
- 30 001 to 50 000 mf/ml
- ≥ 50 001 mf/ml.

A person not involved in the treatment or follow up of the subjects will generate three randomization lists i.e. pairs of randomization number and treatment assignment, one for each of the three strata.

Given the low prevalence of subjects with LLM ≥30001 and even more so ≥50001, the number of subjects in these microfilaremia strata is likely to be small. To ensure equal distribution of the subjects in each microfilaremia stratum across the three treatment arms, randomization will be performed in blocks of three, i.e. among three success randomization numbers, one will be assigned to placebo treatment, one to 2 doses of albendazole and one to 6 doses of albendazole.

Subjects who have qualified and have been selected for the study will be assigned the next unassigned number on the randomization list for their stratum in the order of their subject number.

6.2.5 Study drug preparation

Based on the randomization lists with their pairs of randomization numbers and treatment assignments for each microfilaria stratum, the pharmacist will prepare prior to each treatment round treatment packages labeled with the randomization number and strata.

6.2.6 Study drug administration and recording

For each subject who received treatment, the following information will be immediately recorded after each treatment in the 'site treatment records':

- subject number
- subject initials

- randomization number on the drug package from which his/her treatment was taken
- initials of the person who administered the drug
- date and time of administration

After each visit that involves treatment, the investigator will provide the site treatment records to the pharmacist who will use them to fill in the drug accountability forms and then provide the site treatment records back to the investigator.

If a treatment package was not used because of subject withdrawal, the treatment package will be returned to the pharmacists.

6.2.7 Accountability for study drug

The PI is responsible for ensuring appropriate accounting for all unused study drugs and all used and empty study drug containers.

Drug accountability records will be maintained by the unblinded pharmacist who prepares the treatment packages based on the randomization lists and the records provided by the investigator (see section 6.2.6).

For both albendazole and placebo the following information needs to be recorded:

- **Drug received**: type of drug (albendazole or placebo), lot number, quantity and date of receipt,
- **Drug administered**: For each administration: date, type (albendazole or placebo) quantity, subject randomization number, subject initials, subject number, initials of person who prepared the drug, initials of person who administered the drug (to be provided to the unblinded pharmacist by the investigator or his designee).

At the termination of the study, a final drug accountability review and reconciliation will be completed, any discrepancies will be investigated, and their resolution will be documented. All unused study drugs will be taken care off as per instructions from Glaxo-SmithKline to the Principal Investigator with copy to the TDR project manager.

6.2.8 Prohibited and permitted prior treatments

Benzimidazole treatment within the previous 12 months is an exclusion criterion.

All other medication, whether prescription, over-the-counter or herbal preparations prior to treatment within this study is permitted.

6.2.9 Prohibited concomitant treatment

Subjects will be advised not to take anti-filarial treatment (Mectizan (ivermectin) or Diethylcarbamazine) or any antihelminthic treatment during the study (including albendazole outside the study treatment). Since the diseases for which ivermectin and DEC are indicated are not prevalent in the study area, it is unlikely that subjects would be prescribed these drugs.

The fact that each follow up is associated with a general medical examination and treatment of common illnesses further reduces the probability of subjects pursuing and getting access to these drugs.

There are no restrictions on other concomitant medication.

At each visit, subjects will be asked about the medication they have taken since the last study drug administration and this will be recorded in the source records and case report form, if available with indication, doses and dates of administration.

7 ASSESSMENT OF EFFICACY

7.1 Efficacy Variables

The efficacy evaluation will be based on the examination of the slides generated from the calibrated blood smears for the determination of the *L. loa* microfilaria counts.

7.1.1 Primary

The primary efficacy variable is the proportion of subjects in each treatment group whose microfilaria count has been sustainably reduced by 50% from the baseline value at any time point after the first dose. A sustainable reduction by 50% is defined as a reduction to 50% of baseline LLM for at least 4 months.

7.1.2 Secondary

Secondary efficacy variables are:

- The proportion of subjects whose microfilaria counts have been reduced sustainably to <8100 mf/ml after the first dose
  - by treatment group and microfilaria strata
  - by treatment group stratified by gender
  - by treatment group stratified by gender and initial microfilaria count
- The percent reduction in microfilaria counts at each time point quantitated via the range, William geometric mean and median
  - by treatment group across all patients
  - by treatment group stratified by initial microfilaria count
  - by treatment group stratified by gender
  - by treatment group stratified by gender and initial microfilaria count
- The evolution of *Loa* parasitemia with time in each treatment group analysed via General Linear Model

7.2 Method and timing of measurement of efficacy parameter

The method of parasitological examination at screening and at each efficacy follow up will be the calibrated blood smear (CBS). This examination will be done by finger prick after a careful cleaning of the finger. The blood will be collected with a 50µl non-heparinized capillary tube, spread on a labeled slide, dried and stained. More details are provided in section 6.1.7.1.

Blood samples for baseline LLM as well as for all efficacy follow up evaluations (at M2, M4, M6, M8, and M10, and at M 14 and M18) will be obtained between 11:00 and 15:00 o'clock.

The date and time of day of the collection of a blood sample will be recorded and visits will be planned in a way that each village is visited at the same time of day throughout the study.

In a previous study determining *L. loa* periodicity in subjects who had developed adverse effects after treatment with ivermectin and in control subjects (Kamgno et al., 2004, Figure 1), the *Loa* parasitemia was relatively stable (variation ≤20%) between 11:00 and 15:00.

Collection of samples between 11:00 and 15:00 in combination with scheduling visits to a particular village for the same time of day will ensure that the results will be comparable between and within the groups and that diurnal variation will not compromise the efficacy analysis.

8 ASSESSMENT OF SAFETY

8.1 Safety Variables

All safety variables will be derived from the adverse events reported by the subjects and/or observed during the clinical evaluations.

The frequency of treatment emergent adverse events will be compared by type of adverse event, severity and relationship to study drug between treatment groups.

Serious adverse events will be tabulated by relationship to study drug and treatment group. Narratives will be provided for each event.

8.2 Methods and timing for assessing adverse events

Follow up for adverse events will be conducted on the day of treatment, at day 3 and day 7 after each treatment as well as at the next treatment or efficacy follow up visit (M2, M4, M6, M8, and M10, and at M 14 and M18. Adverse events will also be determined through the clinical examination conducted prior to each treatment, and if clinically indicated through laboratory evaluations. If clinically indicated or indicated based on the report of adverse event(s) by the subject, a complete blood count and laboratory evaluations will be performed as appropriate.

Any study related adverse events will be treated either in the village by the study team or in a hospital or treatment center to which the subject will be transferred if the adverse event cannot be treated in the village by the study team. A district medical officer is part of the study team.

If any non study related adverse events are diagnosed during the clinical examination the subjects will either be treated by the study team if this is within the capacity of the study team or they will be referred to a hospital or treatment center.

In both cases the investigator will discuss with the subjects their continued participation in the study. If this is not consistent with the well being of the subject, the investigator will withdraw the subject from further treatment or from the whole study.

For each adverse event the following information will be recorded:

- Subject number, subject initials, randomization number
- Type of adverse event
- Start date and time
- End date and time
- Severity (see section 8.4)
- Whether the adverse event is due to a pre-existing condition (ie, an acute condition present at the start of the study or history of a chronic condition), and was present prior to study start or study treatment
- Relationship to study treatment (see section 8.5).
- Action taken;
- Outcome.

All adverse events will be followed up to resolution or until a study-unrelated cause has been established.

The methods for clinical examination and laboratory tests for ALT, AST, creatinine and haemoglobin and pregnancy are described in section 6.1.7.2 and 6.1.7.3.

Other laboratory tests will be conducted if clinically indicated.

8.3 Adverse event definitions

8.3.1 Adverse event

An ***adverse event*** is defined as any untoward, undesired, unplanned clinical event in the form of signs, symptoms, disease, or laboratory or physiological observations occurring in a human being participating in a clinical study with a study drug, regardless of causal relationship. This includes the following:

- Any clinically significant worsening of a preexisting condition.
- Any recurrence of a preexisting condition.
- An AE occurring from overdose of a study drug whether accidental or intentional (ie, a dose higher than that prescribed by a health care professional for clinical reasons).
- An AE occurring from abuse of a study drug (ie, use for nonclinical reasons).
- An AE that has been associated with the discontinuation of the use of a study drug.

Note: A procedure is not an AE, but the reason for a procedure may be an AE.

8.3.2 Treatment emergent adverse events

Treatment emergent adverse events are adverse events that occur after administration of the study drug.

8.3.3 Serious adverse event

A ***serious adverse event*** (SAE) is any AE occurring at any dose that meets 1 or more of the following criteria:

- Results in death
- Is life threatening
  The term 'life-threatening' refers to an event in which the subject was at risk of death at the time of the event. It does not refer to an event, which hypothetically might have caused death, if it were more severe.
- Requires inpatient hospitalization or prolongation of an existing hospitalization
  In general, hospitalization signifies that the subject has been detained (usually involving at least one overnight stay) at the hospital or emergency ward for observation and/or treatment that would not have been appropriate in the physician's office or out-patient setting. Complications that occur during hospitalization are AEs. If a complication prolongs hospitalization or fulfills any other serious criteria, the event is 'serious'. When in doubt as to whether 'hospitalization' occurred or was necessary, the AE should be considered 'serious'. Hospitalisation for elective treatment of a pre-existing condition that did not worsen from baseline is not considered an AE.
- Results in a disability / incapacity
  The term disability means a substantial disruption of a person's ability to conduct normal life functions. This definition is not intended to include experiences of relatively minor medical significance such as uncomplicated headache, nausea, vomiting, diarrhoea, influenza and accidental trauma (e.g. sprained ankle) which may interfere or prevent everyday life functions but do not constitute a substantial disruption.
- Results in a congenital anomaly or birth defect

If there is any doubt if an event is an AE or SAE, the event is to be classified as an SAE.

8.4 Assessment of severity of adverse events

The severity of adverse events will be graded as

Mild:	An event that is easily tolerated by the subject, causing minimal discomfort and not interfering with everyday activities
Moderate:	An event that is sufficiently discomforting to interfere with everyday activities
Severe:	An event that prevents normal everyday activities
Not applicable:	Events where intensity is meaningless or impossible to determine (i.e. blindness and coma).

8.5 Assessment of relationship of adverse event to study drug

When assessing the relationship between administration of a study drug and an AE, the following should be considered:

- Temporal relationship between administration of the study drug and the AE
- Biological plausibility of relationship (i.e. consistent with the pharmacological action of the drug)
- Subject's underlying clinical state (concurrent illness, relevant medical history) or concomitant agents and/or therapies
- When applicable, whether the AE decreases in severity on discontinuation of the study drug (dechallenge)
- When applicable, whether the AE reappears or increases in severity on repeat exposure to the study drug (rechallenge, Note: a subject should not be re-administered a drug if there is a suspicion that the drug may have caused an adverse event and there is no reason to believe that the benefit for the patient from the rechallenge outweighs the potential risk of the AE if it is indeed causally related to the drug administration)

**Reasonable possibility of relationship to study drug**	**Criteria**<br>**The adverse event**
**Yes**	Has a reasonable temporal association with study drug administration<br><br><u>And/or</u> follows a known response pattern to the study drug<br><br><u>And</u> could or could not have been produced by other factors such as the subject's clinical state, therapeutic intervention or concomitant therapy
**No**	The event was <u>most probably produced by other factors</u> such as the subject's clinical state, therapeutic intervention or concomitant therapy<br><br>OR<br><br>The event is <u>clearly related to other factors</u> such as the subject's clinical status therapeutic intervention or concomitant therapy

8.6 Reporting of serious adverse events and pregnancies to GSK, TDR and National regulatory authorities

All AEs and SAEs will be recorded on source documents and recorded on CRFs.

8.6.1 Serious adverse events

All SAEs will be reported by the PI on the SAE reporting forms to GSK, and to the National regulatory authorities as per their requirements within 24 hours of the investigator learning about them. The investigator has to provide follow-up information concerning the outcome independent on whether the subject was discontinued from the study due to the pregnancy or other reasons.

The SAE reporting forms in the Attachment include the information to be provided. The filled out forms will be faxed to:

**GSK Global Clinical Safety & Pharmacovigilance (GCSP):**	**FAX**	**+44-208-4239534**
	**PHONE:**	**+44-208-4223434**

8.6.2 Pregnancies

All pregnancies that occur during the study will be reported to GSK within 2 weeks of the investigator learning about them on the form provided in the Attachments.

When possible, all reports of pregnancy must be followed up for information about the course of the pregnancy and delivery, as well as the condition of the newborn and the data reported to GSK on the form provided in the Attachments. The filled out forms will be faxed to the same fax number as the SAE information.

9 DATA ANALYSIS

9.1 Sample Size Justification

A positive response in a subject is defined as a drop in LLM from baseline values by ≥50% lasting at least 4 months. Assuming that in the population less than $1/10^6$ subjects will have a positive response if randomized to the placebo group and at least 50% of the subjects will have a positive response if randomized to the 6 dose albendazole treatment regimen, a sample size of 16 subjects in each treatment arm will provide at least 90% power to detect statistically significant (two-sided $\alpha=0.025$) differences between the placebo group and the 6 dose albendazole treatment group. The same assumptions were made for the 2 dose albendazole treatment regimen so that the same power and sample size considerations apply to the comparison between placebo and the 2 dose albendazole treatment. An alpha value of 0.025 was chosen since two comparisons (placebo vs. 2 doses of albendazole, placebo vs. 6 doses of albendazole) will be performed. Based on prior experience, a drop out rate of approximately 20% is expected, resulting in a sample size of 20 subjects for each of the three treatment arms.

9.2 Statistical Methods

9.2.1 Efficacy analysis

Proportions of patients with a sustained reduction of LLM from baseline by ≥50% between each active treatment group and the placebo group will be compared by the Chi square test.

Loa parasitaemia will be compared at each point between groups using non-parametric test due to the non normal distribution of the *Loa* microfilaremia. The General Linear Model (GLM) will also be used for the comparison of the evolution of *Loa* parasitemia in different groups.

9.2.2 Safety analysis

Calculation of the proportion of subjects with AEs and SAE by treatment group, type of AE, gender, severity and relationship to study drug (see section 8.1).

9.2.3 Analysis populations

Two populations will be analyzed for efficacy:

Per protocol population: includes all subjects who were received the full treatment course they were randomized to and have a final efficacy assessment. .

Intent to treat population: includes all subjects who received at least one dose of study drug and have a final efficacy assessment.

All subjects who received at least one dose of study drug will be included in the safety analysis.

9.2.4 Interim Analysis

No interim analysis is planned.

9.2.5 Reasons for discontinuation of treatment/study

For subjects who have provided reasons for discontinuation of participation in study treatments or the whole study or for their not attending all scheduled treatment days, the reasons will be categorized into four groups:

- reasons related to drug administration,
- reasons related to study drug procedures, but not drug administration,
- other reasons,
- no reason provided

and the proportion of subjects in each category will be calculated by number of treatments prior to withdrawal.

This analysis can serve as input into the design of a follow up study (if the present study provides proof of concept) which needs to include addressing how a multiple dose albendazole regimen can be implemented for community treatment in a way that maximizes compliance. Compliance will be critical to achieving reliable reduction of *Loa loa* microfilaremia below the risk level in the total population to be treated subsequently with ivermectin.

10 PROBLEMS ANTICIPATED

Problems anticipated and contingency plans:

- Prevalence of *L.loa* in the study area and/or percentage of subjects eligible and willing to participate in the study too low to recruit the target number of subjects. This will necessitate screening a larger number of subjects to achieve the necessary number of subjects.
- The number of subjects with high microfilaria levels (>30000) tends to be low, even in areas with high prevalence of *Loa loa*. Since subjects with this level of *Loa loa* microfilaraemia are those most likely to experience signs and symptoms and potential long term sequelae of loiasis and are at high risk for SAE in case of ivermectin treatment, they are of particular interest in this study. If after final subject qualification the number of subjects with >30000 mf/ml is lower than 15, measures for recruitment of subjects with high microfilaria levels will be discussed with the sponsor.

11 ETHICS

11.1 Informed consent

The method for obtaining informed consent is described in section 6.1.3.2. The informed consent information is provided in the Attachment.

11.2 Gender related issues

Ideally, 50% of the subjects in each microfilaria strata would be women. However, because of (1) pregnancy being an exclusion criterion and (2) prevalence and intensity of infection tending to be lower in females than males, it is unlikely that this will be achieved. There is currently no reason to suspect that the host gender affects the response of the parasite to albendazole.

11.3 Potential risks to subjects

No experimental procedure is being used in this study. The protocol procedures the subjects will undergo include a finger prick (to obtain samples for the parasitological examinations at screening, prior to each treatment and at the additional efficacy follow up visits) and a venous blood sample (4ml **)** for determination of ALT, AST, creatinine and haemoglobin levels and a CBC at the screening visit (see section 6.1.3). The risks associated with these procedures are minimal.

Subjects will be treated with albendazole which is being used in Cameroon and other countries for treatment of intestinal worms and has been proven to be safe for mass treatment of lymphatic filariasis without medical supervision (Horton et al., 2000; Ismail et al., 2001). During the previous trials with albendazole in subjects with loiasis, there were either no clinical adverse effects and no observed hepatotoxicity, renal toxicity, or hematologic abnormalities attributable to the drug (Klion *et al.*, 1993) or adverse events were not severe, transient and/or observed with similar frequency in the placebo group (see section 3).

11.4 Potential benefits to subjects

Subjects who participate in the preliminary survey as well as in final subject selection will benefit from having their health status evaluated and either treated immediately, or being referred to a hospital/treatment center for treatment of illnesses that go beyond the treatment capability of the study team in the village.

Any subject who qualifies for and participates in the study will benefit from having clinical examinations and treatment of minor illnesses as part of the study, or referral to the next health center for illnesses outside the scope of village-based treatment during this study every two months.

The prevalence of intestinal helminths, in particular hookworm (Ratard et al, 1992), in the study area is low with less than 15% of adults estimated to be infected. Thus, only the rare subject who participates in the study and has intestinal helminths not accompanied by signs and symptoms (e.g. diarrhoea, abdominal pain, vomiting) and is randomized to one of the two albendazole treatment groups could benefit from the effect of albendazole on their intestinal helminths. Given that albendazole is a very safe treatment, this potential benefit will be extended to subjects who received only placebo during the study by offering them albendazole treatment after completion of the study and unblinding.

If one or both of the albendazole treatment regimens result in reduction of *L.loa* microfilaremia, those subjects in which this reduction occurs will benefit from this.

Since this is a small proof-of-concept study, expansion of a treatment regimen for which the benefit has not yet been definitively established to all study participants or the community from which they are coming is premature.

11.5 Potential benefits to communities

Communities with loiasis would benefit from the development of a safe and effective treatment reducing LLM, because there is currently no safe treatment for to reduce LLM and physicians are thus not able to offer treatment to *Loa loa* infected subjects who asked for that type of treatment.

Among communities with loiasis, those co-endemic for onchocerciasis and/or lymphatic filariasis would benefit additionally from a mass treatment suitable *Loa loa* treatment through the reduced risk of SAEs during CDTI and/or implementation of lymphatic filariasis control.

11.6 Inclusion of a placebo arm

As outlined in section 3, inclusion of a placebo group is necessary to be able to assess whether any reduction of microfilaremia counts in either of the two albendazole treatment arms is statistically significantly associated with the use of albendazole and thus likely to reflect more than the natural longitudinal variation of LLM as seen in the placebo group.

11.7 Data safety monitoring

Given albendazole's excellent safety record in the treatment of intestinal helminths and in lymphatic filariasis control programs, a data safety monitoring board is not required.

11.8 Information of the communities of the study results

Once the final data from the study are available and have been discussed with the stake holders, each community will be visited to inform them about the results and what will be done in consequence of the results obtained.

11.9 Subject Compensation

Subjects included in this trial will be compensated for any expenses incurred during the transport to the study site but will not have direct financial compensation for study participation. Information on indirect compensation via the treatment of minor complaints detected during the clinical examinations is provided in section 11.4.

11.10 Insurance

A clinical trial insurance will be taken out through TDR.

12 OBLIGATIONS OF THE INVESTIGATOR

The principal investigator will ensure that the study will be conducted in accordance with the ethical principles that have their origin in the Declaration of Helsinki (as amended, October 2000) and that are consistent with good clinical practice (GCP) and the Standard Operating Procedures for Clinical Investigators (TDR/TDP/SOP/99.1).

12.1 Ethics Committee Approval

The protocol and the informed consent form will be approved by the Ethics Committee of the Cameroon Health Service and the WHO prior to initiation of the study.

12.2 Pre study Documentation

The principal investigator will provide the sponsor with the following documents:

- A copy of the National Ethics Committee (NEC) approval letter for the protocol and informed consent. Any changes in this study or unanticipated problems involving risks to the subjects will be reported promptly to the NEC and the TDR Product Manager.
- Current signed and dated curricula vitae for the investigators
- Copy of the approved informed consent documents to be used.
- Copy of the protocol sign-off page signed by the investigators.

Subject screening will not be initiated until clearance has been received from the National Ethics Committee and the WHO Ethics Review Committee.

12.3 Study management

The principal investigator is responsible for assigning the responsibilities to each study team member and to ensure that they fulfill their responsibilities.

The study team will consist of:

- One medical doctor who will assist the principal investigator in examining the patients.
- Two Laboratory technicians who will be responsible for collecting blood samples for CBS and laboratory evaluations.
- One nurse who will measure blood pressure and body temperature as needed.
- One secretary who will register patients during the preliminary survey, and who will also help in registering data.
- One local person in each village who will assist the principal investigator in the sensitization of patients and help participants to liaise with the PI during the course of the study between visits to the villages.
- One district medical officer.

12.4 Informed Consent

Informed consent will be obtained prior to any study related procedures (see attached).

12.5 Serious Adverse Event Reporting

The principal investigator agrees to report all SAEs to GSK and the National Cameroonian authority, as per their requirements and described in section 8.6.

**GSK Global Clinical Safety & Pharmacovigilance (GCSP):**	**FAX**	**+44-208-4239534**
	**PHONE:**	**+44-208-4223434**

The principal investigator is responsible for ensuring that any sub-investigator promptly brings SAEs to the attention of the investigator.

12.6 Source Records

The principal investigator is responsible for recording of all relevant data in the source records and their correct transcription to the CRF.

The principal investigator agrees that qualified representatives of the sponsor and the national authority have the right, both during and after this study, to direct access to the source documents.

12.7 Protocol Amendment

Once the study has started, the investigator will adhere to the protocol and ensure that it is strictly followed. Deviations to protocol procedure(s) that involve the subjects will not be made without the agreement of the sponsor except when necessary to avoid immediate danger to a trial subject.

Proposed changes will be discussed with the TDR Product Manager. If the TDR Product Manager agrees to the changes, they will be outlined as a protocol amendment along the lines indicated in the TDR SOP for Clinical Investigators. The document will be signed by the investigator and TDR Product Manager and appended to the original protocol.

Any such modification of the protocol that has an impact on the risk and benefits of the study participants will require a submission to the National EC and the WHO ERC prior to implementation.

In the case of minor modifications that do not have impact on the risk-benefit of the subjects during participation in the trial, or which only impact on administrative activities, the modification will be considered a simple notification that does not require Ethics Committee approval.

12.8 Quality Assurance

The principal investigator is responsible for ensuring that all members of the study team are trained on the requirements for conducting the study according to Good Clinical Practice and the applicable regulatory requirements.

The investigator will accordingly ensure that

- the data in the source records are accurate, complete and legible,
- transcribed accurately to the Case Report Forms
- entered accurately into the data base and analysed as planned in the protocol.

Adherence to GCP, consistency of the data in the source records with those on the CRF and drug accountability will be monitored by a TDR clinical monitor.

12.9 Interim Report

11 months after the initiation of the study the investigator will provide an interim report including a summary of number of subjects enrolled by microfilaria strata, summary statistics of microfilaria counts pre-treatment and at each follow up time point completed up to 1 month prior to the date of the report (range, median, Williams geometric means for each microfilaria stratum), adverse events (number of adverse events by type, severity, seriousness separately for each microfilaria stratum).

The same type of report will be generated 23 months after the initiation of the study unless at that time the study has been completed and a final study report (see section 12.10) has or will shortly be provided within the next 2 months.

12.10 Final Study Report

The clinical investigator will complete a report notifying the sponsor and the national authorities, if required, of the conclusion of the study within 2 weeks of the last follow up visit. A final report including all data analyses and conclusions will be sent to the TDR Product Manager within 3 months of the last follow up visit.

12.11 Record retention

The investigator will retain all study related records until 5 years after the publication of the results unless otherwise notified by the sponsor.

12.12 Change in Investigator(s)

If the principal investigator retires, relocates, or otherwise withdraws from conducting the study, the responsibility for maintaining records will be transferred to another investigator with the same qualifications. The sponsor and the National Ethics Committee will be notified of the change.

12.13 Confidentiality

All unpublished information that the sponsor gives to the investigators will be kept confidential and shall not be published or disclosed except to study personnel under the supervision of the investigator, Ethical Committees, or duly authorized representatives of regulatory agencies, under the condition that they maintain confidentiality. The investigator will not make a patent application based on the results of this study and will not assist any third party in making such an application without the written authorization of the sponsor, unless otherwise specified in the TSA.

13 TERMINATION OF THE STUDY

13.1 Termination by the Sponsor

The sponsor may terminate the study at any time for any of the following reasons:

1. Failure to enroll subjects.
2. Protocol violations.
3. Unsafe or unethical practices.
4. Administrative decision.

13.2 Termination by the Investigator

If the investigator terminates the study prematurely, the investigator will provide a written statement to the sponsor and the National EC describing why the study was terminated prematurely. The investigator will generate a report of the data obtained until the date of study termination.

14 QUALITY ASSURANCE

See section 12.8.

15 PUBLICATIONS

The investigators will target publication of the results in a recognized (refereed) scientific journal. The investigators will submit publications, reports, abstracts, manuscripts and/or other presentation materials to the sponsor for review prior to submission for publication or presentation. The sponsor shall have 60 calendar days to respond with any requested revisions, including without limitation, the deletion of confidential information. The investigators will act in good faith upon such requested revisions, except the investigators will delete any confidential information from such proposed publication or presentation.

16 REFERENCES

Awadzi K, Hero M, Opoku NO, Buettner DW, Coventry PA, Prime MA, Orme M.L'E, Edwards G. (1994) The chemotherapy of Onchocerciasis XVII. A clinical evaluation of albendazole in patients with onchocerciasis; effects of food and pretreatment with ivermectin on drug response and pharmacokinetics. Trop. Med Parasitol 45, 203-208.

Blum J, Wiestner A, Fuhr P, Hatz C (2001) Encephalopathy follow Loa loa treatment with albendazole. Acta Tropic 78: 63-65.

Boussinesq, M., Gardon, J., Gardon-Wendel, N., and Chippaux, J.P. (2003) Clinical picture, epidemiology and outcome of Loa-associated serious adverse events related to mass ivermectin treatment of onchocerciasis in Cameroon. Filaria J 2 Suppl 1: S4.

Boussinesq, M., Gardon, J., Gardon-Wendel, N., Kamgno, J., Ngoumou, P., and Chippaux, J.P. (1998) Three probable cases of Loa loa encephalopathy following ivermectin treatment for onchocerciasis. Am J Trop Med Hyg 58: 461-469.

Ducorps PM, Gardon/Wendel N, Ranque S, Ndong W, Boussinesq M, Gardon J, Schneider D, Chippaux JP (1995) Effets secondaires du traitement de la loase hypermicrofilaremique par l'ivermectine. Bull Soc Path Ex 88, 105-112

Duke, BOL (1960) Studies on loiasis in monkeys. II - The population dynamics of the microfilariae of Loa in experimentally infected drills (Mandrillus leucophaeus). Nnals of Tropical Medicine and Parasitology 54, 15-31.

Duke, BOL (1991) Loiasis. In: Strickland GT (Ed) Hunter's Tropical Medicine, WB Saunders Company, Philadelphia, London, Toronto, Montreal, Sydney, Tokyo, 7th edition, pp727-729.

Gardon, J., Kamgno, J., Folefack, G., Gardon-Wendel, N., Bouchite, B., and Boussinesq, M. (1997) Marked decrease in Loa loa microfilaraemia six and twelve months after a single dose of ivermectin. Trans R Soc Trop Med Hyg 91: 593-594.

Glaxo Smith Kline 2001, Albenza prescribing information

Horton J (2000) Albendazole: a review of anthelminthic efficacy and safety in humans. Parasitology 121, Supplement, S113-S132.

Horton, J., Witt, C., Ottesen, E.A., Lazdins, J.K., Addiss, D.G., Awadzi, K. et al. (2000) An analysis of the safety of the single dose, two drug regimens used in programmes to eliminate lymphatic filariasis. Parasitology 121 Suppl: S147-160.

Ismail, M.M., Jayakody, R.L., Weil, G.J., Fernando, D., De Silva, M.S., De Silva, G.A., and Balasooriya, W.K. (2001) Long-term efficacy of single-dose combinations of albendazole, ivermectin and diethylcarbamazine for the treatment of bancroftian filariasis. Trans R Soc Trop Med Hyg 95: 332-335.

Kamgno, J., and Boussinesq, M. (2002) Effect of a single dose (600 mg) of albendazole on Loa loa microfilaraemia. Parasite 9: 59-63.

Kamgno, J.Twum-Danso N. A., Boussinesq, M., Duke, B. O. L. and Thylefors, B. Do specific strains of *Loa loa* influence the occurrence of post-ivermectin *Loa* encephalopathies? Comparison of the patterns of *Loa loa* microfilarial periodicity in patients who developed severe adverse events and in matched controls. *32nd Meeting of the Mectizan® Expert Committee / Albendazole Coordination, Atlanta 28-30 April 2004.*

Klion, A.D., Massougbodji, A., Horton, J., Ekoue, S., Lanmasso, T., Ahouissou, N.L., and Nutman, T.B. (1993) Albendazole in human loiasis: results of a double-blind, placebo-controlled trial. J Infect Dis 168: 202-206.

Makunde WH, Kamugisha LM, Massaga JJ, Makunde RW, Xavael ZX, Akida J, Salum FM, Taylor MJ, 2003, Treatment of co-infection with bancroftian filariasis and onchocerciasis: a safety and efficacy study of albendazole with ivermectin compared to treatment of single infection with bancroftian filariasis. Filaria Journal 2: 15-24.

MDP (1996): Central Nervous System (CNS) complications of loiasis and adverse CNS events following treatment: Report of an invited consultation, 2-3 October 1995.

Mectizan Expert Committee (2002) Report of a scientific working group on serious adverse events following Mectizan treatment of onchocerciasis in *Loa loa* endemic areas. July 2002

Mectizan Expert Committee and The Technical Consultative Committee, 2004, Recommendations for the treatment of onchocerciasis with MEctizan in areas co-endemic for onchocerciasis and Loiasis.

Pani SP, Subramanyam Reddy G, Das LK, Vanamail P, Hoti SL, Ramesh J, Das PK, 2002 Tolerability and efficacy of single dose albendazole, diethylcarbamazine citrate (DEC) or co-administration of albendazole with DEC in the clearance of *Wuchereria bancrofti* in asymptomatic microfilaraemic volunteers in Pondicherry, South India: a hospital-based study. Filaria Journal 1: 1-11.

Parry E, Godfrey R; Mabey D, Gill G (2004) (Eds). Principles of Medicine in Africa. Cambridge University Press, pp 431-433.

Ratard RC, Kouemeni LE, Ekani Bessala MK, Ndamkou CN (1992) Distribution of hookworm infection in Cameroon. Ann Trop Med Parasitol 86: 413-418.

Tabi, T.E., Befidi-Mengue, R., Nutman, T.B., Horton, J., Folefack, A., Pensia, E. et al. (2004) Human loiasis in a Cameroonian village: a double-blind, placebo-controlled, crossover clinical trial of a three-day albendazole regimen. Am J Trop Med Hyg 71: 211-215.

Takougang, I., Meremikwu, M., Wandji, S., Yenshu, E.V., Aripko, B., Lamlenn, S.B. et al. (2002) Rapid assessment method for prevalence and intensity of Loa loa infection. Bull World Health Organ 80: 852-858.

Thomson, M.C., Obsomer, V., Kamgno, J., Gardon, J., Wanji, S., Takougang, I. et al. (2004) Mapping the distribution of Loa loa in Cameroon in support of the African Programme for Onchocerciasis Control. Filaria J 3: 7.

Tsague-Dongmo, L., Kamgno, J., Pion, S.D., Moyou-Somo, R., and Boussinesq, M. (2002) Effects of a 3-day regimen of albendazole (800 mg daily) on Loa loa microfilaraemia. Ann Trop Med Parasitol 96: 707-715.

Twum-Danso, N.A. (2003a) Loa loa encephalopathy temporally related to ivermectin administration reported from onchocerciasis mass treatment programs from 1989 to 2001: implications for the future. Filaria J 2 Suppl 1: S7.

Twum-Danso, N.A. (2003b) Serious adverse events following treatment with ivermectin for onchocerciasis control: a review of reported cases. Filaria J 2 Suppl 1: S3.

Twum-Danso, N.A. (2006) personal communication

WHO, 2003, Report on active surveillance for adverse events following the use of drug co-administrations in the Global Programme to Eliminate Lymphatic Filariasis, Weekly Epidemiological Records 35: 315-317.

17 ATTACHMENTS

Informed Consent Information for Initial Screening (preliminary survey)

Informed Consent Information for final subject qualification and study participation

SAE Reporting Form

SAE Form (Section 12, for follow up information)

Pregnancy Notification Form

Pregnancy Follow-Up Form - Current Pregnancy Information

Protocol Amendment

Efficacy of albendazole in decreasing *Loa loa* microfilaraemia

The protocol of the study on the Efficacy of albendazole in decreasing *Loa loa* microfilaraemia started in March 2007 in the south Province of Cameroon. This study which was a double blind study had to continue until 18 month (September 2008). The study continued to this term and the blind was lifted. Due to tendency of decrease in one of the group, it was proposed that the follow-up should continue to 24 months with two supplementary control of the *Loa loa* microfilaremia at M21 and M24.

The procedure of blood sampling will remain as from M0 to M24. The blood sample will be collected almost and the same time, with the same material and with the same precautions. The examination of slides will follow the same procedures as the previous examinations.

Signing of informed consent.

Patients of the present trial signed an informed consent to participate at the study up to 18 months. This trial was prolonged to 24 months. We will propose another informed consent to patients for the prolongation of the study. The objectives of this prolongation will be explained to all the patients and they will sign the third informed consent. They will be examined for the two last calibrated blood smears as at M14 and M18.

Subject Compensation

Subjects who accept to continue the study and who signs the informed consent will be compensated for any expenses incurred during the transport to the study site but will not have direct financial compensation for study participation.

Information of the subjects of the study results

During explanation for the continuation of the study, the investigators will explain the tendency of the LLM of each patient. This explanation will be completed when the final data from the study will be available. Each patient will be informed individually about the evolution of his *Loa loa* microfilaremia.
